# Comparison of two cash transfer strategies to prevent catastrophic costs for poor tuberculosis-affected households in low- and middle-income countries: An economic modelling study

**DOI:** 10.1371/journal.pmed.1002418

**Published:** 2017-11-07

**Authors:** William E. Rudgard, Carlton A. Evans, Sedona Sweeney, Tom Wingfield, Knut Lönnroth, Draurio Barreira, Delia Boccia

**Affiliations:** 1 Department of Infectious Disease Epidemiology, London School of Hygiene & Tropical Medicine (LSHTM), London, United Kingdom; 2 Innovation For Health And Development (IFHAD), Section of Infectious Diseases & Immunity, Imperial College London and Wellcome Trust Imperial College Centre for Global Health Research, London, United Kingdom; 3 Innovación Por la Salud Y Desarrollo (IPSYD), Asociación Benéfica PRISMA, Lima, Perú; 4 Innovation For Health And Development (IFHAD), Laboratory of Research and Development, Universidad Peruana Cayetano Heredia, Lima, Perú; 5 Department of Global Health and Development, London School of Hygiene & Tropical Medicine (LSHTM), London, United Kingdom; 6 Infectious Diseases & Immunity, Imperial College London, and Wellcome Trust Imperial College Centre for Global Health Research, London, United Kingdom; 7 Tropical and Infectious Diseases Unit, Royal Liverpool and Broadgreen University Hospital, Liverpool, United Kingdom; 8 Institute of Infection and Global Health, University of Liverpool, Liverpool, United Kingdom; 9 Department of Public Health Sciences, Karolinska Institutet, Stockholm, Sweden; 10 National TB Control Program (NTP), Secretariat of Health Surveillance, Ministry of Health of Brazil, Brasília DF, Brazil; University of California San Francisco, UNITED STATES

## Abstract

**Background:**

Illness-related costs for patients with tuberculosis (TB) ≥20% of pre-illness annual household income predict adverse treatment outcomes and have been termed “catastrophic.” Social protection initiatives, including cash transfers, are endorsed to help prevent catastrophic costs. With this aim, cash transfers may either be provided to defray TB-related costs of households with a confirmed TB diagnosis (termed a “TB-specific” approach); or to increase income of households with high TB risk to strengthen their economic resilience (termed a “TB-sensitive” approach). The impact of cash transfers provided with each of these approaches might vary. We undertook an economic modelling study from the patient perspective to compare the potential of these 2 cash transfer approaches to prevent catastrophic costs.

**Methods and findings:**

Model inputs for 7 low- and middle-income countries (Brazil, Colombia, Ecuador, Ghana, Mexico, Tanzania, and Yemen) were retrieved by literature review and included countries' mean patient TB-related costs, mean household income, mean cash transfers, and estimated TB-specific and TB-sensitive target populations. Analyses were completed for drug-susceptible (DS) TB-related costs in all 7 out of 7 countries, and additionally for drug-resistant (DR) TB-related costs in 1 of the 7 countries with available data. All cost data were reported in 2013 international dollars ($). The target population for TB-specific cash transfers was poor households with a confirmed TB diagnosis, and for TB-sensitive cash transfers was poor households already targeted by countries’ established poverty-reduction cash transfer programme. Cash transfers offered in countries, unrelated to TB, ranged from $217 to $1,091/year/household. Before cash transfers, DS TB-related costs were catastrophic in 6 out of 7 countries. If cash transfers were provided with a TB-specific approach, alone they would be insufficient to prevent DS TB catastrophic costs in 4 out of 6 countries, and when increased enough to prevent DS TB catastrophic costs would require a budget between $3.8 million (95% CI: $3.8 million–$3.8 million) and $75 million (95% CI: $50 million–$100 million) per country. If instead cash transfers were provided with a TB-sensitive approach, alone they would be insufficient to prevent DS TB-related catastrophic costs in any of the 6 countries, and when increased enough to prevent DS TB catastrophic costs would require a budget between $298 million (95% CI: $219 million–$378 million) and $165,367 million (95% CI: $134,085 million–$196,425 million) per country. DR TB-related costs were catastrophic before and after TB-specific or TB-sensitive cash transfers in 1 out of 1 countries. Sensitivity analyses showed our findings to be robust to imputation of missing TB-related cost components, and use of 10% or 30% instead of 20% as the threshold for measuring catastrophic costs. Key limitations were using national average data and not considering other health and social benefits of cash transfers.

**Conclusions:**

A TB-sensitive cash transfer approach to increase all poor households’ income may have broad benefits by reducing poverty, but is unlikely to be as effective or affordable for preventing TB catastrophic costs as a TB-specific cash transfer approach to defray TB-related costs only in poor households with a confirmed TB diagnosis. Preventing DR TB-related catastrophic costs will require considerable additional investment whether a TB-sensitive or a TB-specific cash transfer approach is used.

## Abstract 2

In this modelling study, William Rudgard of the London School of Hygiene and Tropical Medicine and colleagues compare two cash-transfer strategies to avert catastrophic costs for poor tuberculosis-affected households in low- and middle-income countries.

## Author summary

**Why was this study done?:**

- Household costs related to active drug-susceptible (DS) or drug-resistant (DR) tuberculosis (TB) disease include costs for consultations, transport to and from clinics, increased food needs and lost income. If these costs are greater than or equal to one-fifth (20%) of the household’s annual income, then the patient is at risk of unsuccessful TB treatment and these high costs are termed “catastrophic costs.”
- The World Health Organization’s End TB Strategy prioritises preventing TB-affected households from facing catastrophic costs and proposes cash transfers as one way to achieve this. However, there are at least 2 approaches by which cash transfers could be provided to TB-affected households. In the first, they are provided to defray/reimburse households’ TB-related costs (termed a “TB-specific” approach). In the second, they are provided to increase households’ pre-illness income to prevent poverty and strengthen their economic resilience (termed a “TB-sensitive” approach).
- Lack of available individual-level data sources has meant that no studies have compared a TB-specific versus a TB-sensitive cash transfer approach. A literature review combined with a secondary data analysis was an effective way to bring together relevant data from several different sources and model the potential of cash transfers provided by these 2 approaches to prevent catastrophic costs.

**What did the researchers do and find?:**

- We performed a rigorous review of public data sources available on the internet, extracting national average data published between 2005 and 2013 for the 7 low- and middle-income economy countries of Brazil, Colombia, Ecuador, Ghana, Mexico, Tanzania, and Yemen. The data values we extracted included the countries’ mean value of TB patient costs, mean household income, mean cash transfers, and the expected size of the population that would be targeted with either TB-specific or TB-sensitive cash transfers. In all 7 countries these analyses were completed for DS TB, and in 1 of the 7 countries we were also able to complete these analyses for DR TB.
- Expressing TB patient costs as a percentage of household income, we found that average DS TB costs were catastrophic in 6 out of the 7 countries included in the study. In these 6 countries, TB-specific cash transfers prevented DS TB catastrophic costs in only 2, whilst TB-sensitive cash transfers did not prevent DS TB catastrophic costs in any of them. In the 1 country with available data, average DR TB costs were catastrophic, and neither TB-specific nor TB-sensitive cash transfers were sufficient to prevent this.
- For both TB-specific and TB-sensitive approaches, we then estimated the total value that cash transfers would need to be increased to in order to prevent the countries’ average DS or DR TB costs from being catastrophic for DS or DR TB-affected household. Based on this, we also estimated the average budget that each country would need to prevent catastrophic costs for all DS or DR TB-affected households. We found that a TB-specific approach was much more affordable than a TB-sensitive approach.

**What do these findings mean?:**

- The potential of cash transfers to prevent TB-related catastrophic costs is greater if they are provided to defray/reimburse poor households’ costs (TB-specific approach) rather than to increase the income and strengthen the economic resilience of poor households with high TB risk (TB-sensitive approach).
- Where cash transfers are insufficient to prevent catastrophic costs, it will be cheaper to supplement their value to achieve this objective using a TB-specific approach rather than a TB-sensitive approach.
- Important study limitations were that the study was at the country-level, so we might have underestimated the potential of cash transfers to prevent catastrophic costs. Also, we did not assess other health and social benefits of cash transfers, so the impact of TB-specific versus TB-sensitive cash transfers was only judged from the perspective of preventing catastrophic costs.

## Introduction

Tuberculosis (TB) disproportionately affects poor households in low- and middle-income countries that are least able to afford the burden that TB-related costs represent relative to their income [1–6]. Even when diagnosis and treatment is available free of direct charges, TB-affected households are known to incur hidden “out of pocket” direct medical costs (e.g., for consultations) and direct nonmedical costs (e.g., for transport, additional food and symptomatic medicines), as well as indirect costs from lost income [7,8]. Combined, these costs can have severe consequences for affected households. They hinder patients’ access to care and increase their odds of adverse TB treatment outcomes, which are abandoning or failing treatment, dying during treatment, or having recurrent TB within 30 months of starting TB treatment [9–15]. They also force some households to engage in damaging financial coping strategies, which sometimes referred to collectively as dissaving, include taking out a loan, selling productive assets, reducing consumption expenditure to below basic needs, taking children out of education, and/or taking out large loans [16]. Two groups of households that are especially vulnerable to TB-related costs are those in the countries’ poorest population quintile and those affected by drug-resistant (DR) TB [7].

Addressing households’ TB-related costs is essential for ensuring that people with active TB disease are able to access TB diagnosis and treatment. Acknowledging this, the World Health Organization’s End TB Strategy includes a high-level financial risk protection milestone for 2020: “zero TB-affected households facing catastrophic costs due to TB” [17,18]. In this milestone, “catastrophic costs” refers to a combination of direct medical, direct nonmedical, and indirect costs excessive enough to increase a patient’s risk of adverse TB treatment outcome and/or force their household to engage in damaging financial coping strategies [19]. By encompassing all 3 cost components, the term “catastrophic costs” is distinct from the term “catastrophic health expenditure,” which only considers direct medical costs and is used to monitor progress towards financial risk protection as part of universal health coverage [19]. As part of the End TB Strategy, research has focussed on developing an empirical measure of catastrophic costs. Recently, total TB-related costs greater than or equal to 20% of TB-affected households’ pre-illness annual income have been found to significantly increase the likelihood of TB patients experiencing an adverse treatment outcome and their household engaging in damaging coping strategies [14,15]. As the only indicator established to be clinically and financially relevant for assessing a household’s ability to pay for TB care, this measurement of catastrophic costs has tentatively been included by the Global TB Programme in a pilot tool to monitor catastrophic costs of TB-affected households worldwide [19].

Preventing catastrophic costs for TB-affected households is a priority for facilitating individuals’ access to TB diagnosis and treatment, increasing their likelihood of treatment success and reducing onwards TB transmission [18]. With this objective, the Global TB Programme endorses social protection initiatives including cash transfers, food baskets, social insurance and labour market measures to complement universal health coverage initiatives like prepayment, resource pooling, and patient-friendly service delivery [19]. In the TB literature, evidence from a randomized trial in Peru shows that when provided as incentives to support TB treatment, cash transfers reduce poor TB-affected households’ likelihood of incurring catastrophic costs, as well as improve patients’ likelihood of TB treatment success, and uptake of preventative therapy amongst people they are in close contact with (e.g., family, friends, care giver) [15,20]. Outside of the TB literature, synthesised evidence from governmental poverty-reduction policies in several low- and middle-income countries provides evidence that cash transfers increase poor households’ income and consumption expenditure, help them cope with livelihood risks (e.g., illness and unemployment), and support family investments in the human capital of their children (e.g., sending them to school and taking them to regular health checks) [21–23].

Currently, there are at least 2 alternative approaches proposed in the TB literature for providing cash transfers to TB-affected households [24]. The first is termed a “TB-specific” approach, whereby cash transfers would be targeted to poor households with a confirmed TB diagnosis to incentivise and enable TB treatment by defraying their TB-related costs [24]. This approach is exemplified by the cash transfer component of the Community Randomized Evaluation of a Socioeconomic Intervention to Prevent TB (CRESIPT) trial in Peru [25,26]. The second is termed a “TB-sensitive” approach, whereby cash transfers would be targeted to poor households at high risk of developing active TB disease to increase their income, thereby protecting them from poverty-related risk factors for TB infection, progression, and adverse treatment outcomes (e.g., poor living conditions and undernutrition), as well as strengthen their economic resilience to TB-related costs [24]. This approach already exists in many low- and middle-income countries, and is exemplified by governmental poverty-reduction cash transfer programmes like Programa Bolsa Familia in Brazil [27,28].

Depending on whether cash transfers are provided with a TB-specific or a TB-sensitive approach, their impact might vary [24]. We aimed to investigate how this might relate to the potential of cash transfers to prevent catastrophic costs.

## Methods

With no known data sources for investigating if the potential of TB-specific and TB-sensitive cash transfers to prevent catastrophic costs varies, we undertook an economic modelling study using published national average data gathered from a rigorous review of the literature. Our economic modelling study was aggregated at the country level. The setting was low- and middle-income countries where over 95% of TB cases live and where formal institutions to protect households from the social and economic impacts of illness are weakest [29]. The intervention being investigated was cash transfers paid to poor households, and the alternative approaches being compared were: (1) cash transfers provided to defray TB-related costs of households with a confirmed TB diagnosis (termed a “TB-specific” approach); versus (2) cash transfers provided to increase income of households with high TB risk and strengthen their economic resilience (termed a “TB-sensitive” approach). These approaches were compared because of current uncertainty about the potential of each approach to prevent catastrophic costs. Using only TB-related costs incurred by patients, study outcomes were assessed from the patient perspective.

Primary study outcomes were an indicator for catastrophic costs after TB-specific versus TB-sensitive cash transfers, and the countries’ country-level cash transfer budget needed to prevent catastrophic costs for each of these approaches. Catastrophic costs were estimated over a time horizon from the onset of TB symptoms to TB treatment completion. The countries’ country-level cash transfer budgets were estimated over a time horizon of 1 year. In the 1 country with available data, outcomes were investigated separately for drug-susceptible (DS) TB and DR TB. The reason for this is that treatment of DR TB versus DS TB is longer and more intensive and is therefore associated with much higher TB-related costs [7]. The study used cross-sectional data drawn from secondary sources. Data inputs were countries’ mean patient TB-related cost, mean pre-illness household income, mean poverty-reduction cash transfer, and TB-specific versus TB-sensitive target populations. Inputs were retrieved by reviewing TB-related cost and cash transfer literature and countries’ national statistics. Because there was insufficient data across low- and middle-income countries on programmes providing cash transfers with a TB-specific approach, this study compared cash transfers offered by existing governmental poverty-reduction programmes as if they were provided with a TB-specific versus a TB-sensitive approach.

For transparency, the study was reported according to the Consolidated Health Economic Evaluation Reporting Standards (CHEERS) checklist [30]. The completed checklist is provided in S1 CHEERS checklist. The study’s prospective analysis plan is provided in S1 Text. In the present analysis, we did not attempt to model the potential of TB-inclusive cash transfers to prevent catastrophic costs, and results from key informant interviews are reported elsewhere [31]. Extraction of cash transfer target population data, estimation of 95% confidence intervals (95% CIs), and our sensitivity analyses were added in the peer review process. Key study definitions are listed in Box 1.

**Box 1. Summary of key study definitions.**

**TB-specific cash transfer:** Assistance in the form of cash to poor households with a confirmed TB diagnosis to defray their TB-related costs and thus enable their access to TB diagnosis and treatment [24].

**TB-sensitive cash transfer:** Assistance in the form of cash to poor households at high risk of developing active TB disease to relieve poverty by increasing their income and strengthening their economic resilience [24].

**Direct costs:** The sum of direct medical costs and direct nonmedical costs [7].

1. *Direct medical costs*: Expenses paid for medical examinations and TB medicines because of TB illness (e.g., consultation fees, hospitalisation fees, and fees for diagnostic tests).
2. *Direct nonmedical costs*: Expenses paid for nonmedical items related to TB illness and care (e.g., patient or guardian transportation, additional food, natural nonprescribed remedies).

**Indirect costs:** Income estimated to be lost due to time off work because of TB illness and care (e.g., patient or guardian lost income) [7].

**Pretreatment costs:** The sum of direct and indirect costs incurred between the onset of TB symptoms and receipt of confirmed TB diagnosis.

**During-treatment costs:** The sum of direct and indirect costs incurred between confirmed TB diagnosis to completion of TB treatment.

**Total costs:** The sum of pre- and during-treatment costs.

**TB-related cost burden:** Total TB-related costs expressed as a percentage of annual household income.

**Catastrophic costs:** A value of total TB-related costs excessive enough to increase a patient’s risk of an adverse TB treatment outcome and/or force them to engage in damaging financial coping strategies (e.g., taking out a loan or selling household items) [14].

**Adverse TB treatment outcome:** Abandoning or failing treatment, dying during treatment, or having recurrent TB within 30 months of starting TB treatment [14].

### Study population

In this study, the target population for cash transfers provided with a TB-specific approach was households in countries’ poorest population quintile with a confirmed TB diagnosis. Guidance is not currently available for which TB-affected households should be targeted with a TB-specific approach. We chose to focus on TB-affected households in countries’ poorest population quintile because they are typically at greater risk of incurring catastrophic costs [14]. Whilst it might have been preferable to focus on all TB-affected households that incur catastrophic costs, at the time of analysis no estimates of the size of this population were available in any countries included in this study. The target population for cash transfers provided with a TB-sensitive approach was households in poverty already targeted by countries’ established governmental poverty-reduction cash transfer programme.

### Data sources

#### TB-related cost data

Data on mean TB-related costs incurred by patients were sourced from articles identified by 2 recent publically available systematic reviews [7,8]. These reviews were chosen because they provided a comprehensive, peer-reviewed list of TB-related cost surveys published before March 2013 and February 2015. TB-related cost surveys in identified articles were eligible if they were conducted in a low- or middle-income country and reported mean total costs calculated from direct costs and/or indirect costs incurred over the full duration of pre- and/or during-DS TB and/or DR TB treatment. We excluded cost surveys only reporting median total costs because of difficulties generalising this measure to countries’ total population. We also excluded cost surveys that were conducted before 2006, the year in which the Global TB Programme recommended that governments should waive direct costs for basic TB diagnostic tests and medicines [32]. If a publication reported TB-related costs from surveys in several different countries, each survey country was checked separately for cash transfer and household income data. Data extracted from eligible TB-related cost surveys comprised: survey country, year of data collection, survey setting, survey sample size, local currency unit exchange rate, methods used to estimate TB-related costs, and mean TB-related costs stratified into subcategories of direct, indirect, and total TB-related costs. In Brazil and Yemen, where articles reported mean TB-related costs for different patient subgroups (e.g., directly observed therapy versus self-administered therapy), an unweighted mean overall estimate was calculated across subgroups [33,34].

#### Cash transfer data

For countries with an eligible TB-related cost survey, existing poverty-reduction cash transfer programmes operating in respective countries were identified using the publically available social safety net program inventory in the appendix of the World Bank Group publication “The State of Social Safety Nets 2015” [35]. None of the identified cash transfer programmes were operated with explicit TB objectives. Cash transfer programmes were eligible if they were directed by a national government with the objective of poverty reduction and promoting family human capital development. Operational data on cash transfer programmes were sourced from the original reference [35] and other publically available online data sources identified from Google by combining the phrase “cash transfer” with the name of the programme and the selected country [36–47]. A summary of cash transfer data sources used in the study is provided in S1 Table. Cash transfer programmes were excluded if they were targeted uniquely to senior citizens or pregnant women. Data extracted on eligible poverty-reduction cash transfer programmes comprised: name of programme, breakdown of cash transfer benefits, mean cumulative annual cash transfers, sample size used to summarise mean cumulative annual cash transfers, and the range of cumulative annual cash transfers based on programme regulations.

#### Household income data

For countries with an eligible TB-related cost survey and existing poverty-reduction cash transfer programme, we used countries’ mean household income or expenditure in the poorest population quintile to approximate household income of both TB-specific and TB-sensitive target populations. Publically available summary estimates of household income or expenditure were identified by searching countries’ national statistical websites and the International Household Survey Network’s (IHSN’s) survey catalogue [48]. We assumed that household income and expenditure were approximately similar. Where available, household income was preferred because of its use in the method included by the Global TB Programme in a pilot tool to measure and monitor catastrophic costs of TB-affected households [19]. Data extracted from countries’ national statistical websites and the IHSN survey catalogue comprised: coverage in households of country household income or expenditure survey, and mean household income or expenditure in countries’ poorest population quintile. When household income data was reported by population decile rather than population quintile, an un-weighted mean overall estimate was recalculated across the two poorest deciles. When national income surveys reported mean monthly or quarterly household income or expenditure, these values were extrapolated to mean annual estimates.

#### Target population data

For countries with an eligible cost survey, cash transfer programme, and household income or expenditure survey, we identified the approximate size of their TB-specific target population using the World Health Organization’s publically available TB data [49]. Because estimates of the percentage of TB-affected households represented in the poorest population quintile were not available in any countries included in the study, we used the unweighted mean multiplier for TB prevalence in the poorest population quintile observed in India and South Africa to estimate the size of countries’ TB-specific target population [50,51]. Therefore, countries’ TB-specific target population was extracted as 40% of the countries’ estimated 2013 DS TB burdens or 2015 DR TB burdens. For country estimates of DR TB burden, we used 2015 estimates because 2013 estimates weren’t available. We assumed that each estimated case of active TB disease in the World Health Organization’s TB data represented 1 household with a confirmed TB diagnosis. We identified the approximate size of countries’ TB-sensitive target population using publically available estimates of countries’ 2013 poverty-reduction cash transfer programme coverage in households already extracted in the cash transfer data literature review [35–46]. Countries’ TB-specific and TB-sensitive target populations were also extracted as a percentage of countries’ total population in households using publically available census data available in the United Nations demographic yearbook [52].

All data were extracted into Microsoft Excel 2016.

### Currency and price date

To allow comparison of monetary data extracted in different currencies and measured in different years, all extracted monetary values were inflated and converted to 2013 international dollars using the purchasing power parity conversion factor that accounts for differences in the cost of living across countries [53,54].

### Data management

In countries that had missing values for direct or indirect costs pre- or during-treatment, we estimated their value. To do this, we assumed that average TB-related costs followed a make-up of cost components equivalent to the one synthesised by Tanimura et al. in their systematic review of TB-related costs in low- and middle-income countries, which is that direct and indirect costs are equivalent to 40% and 60% of total costs respectively, and pre- and during-treatment costs are each equivalent to 50% of total costs respectively [7]. Because only 1 included cost survey reported the standard deviation of total costs [33], we also assumed that average TB-related costs had a standard deviation with the same ratio to total costs as the one estimated by Tanimura et al. for average total costs across all low- and middle-income countries, which was 1.1 [7]. We used the assumed standard deviation and the sample size of countries’ cost surveys to calculate 95% CIs for estimated TB-related costs.

### Data analysis

All analyses used published mean national data. To account for uncertainty in the value of extracted TB-related costs, annual household income, and cash transfers, we conducted a multiway analysis that allowed all 3 of these inputs to vary simultaneously according to their sampling distributions. Sampling distributions were simulated from 10,000 computationally generated random samples and were all assumed to have normal distributions according to the central limit theorem. Random samples were generated for TB-related costs using a standard deviation with a ratio of 1.1 to mean estimates, which was the ratio estimated by Tanimura et al. for average total costs across all low- and middle-income countries, and a sample size equivalent to countries’ cost surveys [7]. For annual household income, we used a standard deviation with a ratio of 0.8 to mean estimates, which was the average observed across 2 studies investigating the household-level income effect of poverty-reduction cash transfer programmes in Brazil and Colombia [37,44] and a sample size equivalent to countries’ household income surveys [55–61]. For cash transfers, we used a standard deviation with a ratio to mean estimates equivalent to a quarter of maximum cash transfers minus minimum cash transfers, and a sample size equivalent to the one reported in studies from which we extracted mean cash transfers. In Ecuador and Ghana, we did not simulate sampling distributions for cash transfers because, respectively, all beneficiary households receive the same flat cash transfer, and the mean cash transfer we extracted was estimated from all beneficiary households. Throughout our analysis, 95% CIs were calculated for model estimates using the quantile method. All analyses were run in R version 3.3.0.

#### Estimation of TB-related cost burden before cash transfers

To estimate if TB-related costs were catastrophic for poor TB-affected households, we calculated each country’s TB-related cost burden without cash transfer data by expressing TB-related costs as a percentage of household income. A TB-related cost burden greater than or equal to 20% was measured as catastrophic, as this threshold has been shown to significantly increase the likelihood of TB patients experiencing an adverse treatment outcome, and their household engaging in damaging financial coping strategies [14,15]. In countries where the TB-related cost burden was estimated to be catastrophic, we then compared the potential of cash transfers provided with a TB-specific versus a TB-sensitive approach to prevent catastrophic costs.

#### Estimation of the potential of TB-specific cash transfers to prevent catastrophic costs

To estimate the potential of cash transfers provided with a TB-specific approach to prevent catastrophic costs, we considered that cash transfers would be targeted to poor households with a confirmed TB diagnosis to defray TB-related costs incurred pre- and during-treatment. Thus, we subtracted the value of cash transfers from TB-related costs and then recalculated countries’ TB-related cost burden (Box 2, Equation 1).

**Box 2. Summary of equations used in data analysis.**

**TB-related cost burden (%)**

**Equation 1:** after TB-specific cash transfers

$$TB\;related\;cost\;burden=\frac{\left(TB\;related\;cost-cash\;transfer\right)}{pre\;illness\;HH\;income}*100$$

**Equation 2:** after TB-sensitive cash transfers

$$TB\;related\;cost\;burden=\frac{TB\;related\;cost}{\left(pre\;illness\;HH\;income+cash\;transfer\right)}*100$$

**Additional cash transfer needed to prevent catastrophic costs ($)**

**Equation 3:** TB-specific approach

(i)

$$20=\frac{\left(TB\;related\;cost-cash\;transfer-additional\;cash\;transfer\right)}{pre\;illness\;HH\;income}*100$$

(ii)

additionalcashtransfer=(TBrelatedcost−cashtransfer)−(preillnessHHincome*0.2)

**Equation 4:** TB-sensitive approach

(i)

$$20=\frac{TB\;related\;cost}{\left(pre\;illness\;HH\;income+cash\;transfer+additional\;cash\;transfer\right)}*100$$

(ii)

additionalcashtransfer=(TBrelatedcost/0.2)−(preillnessHHincome+cashtransfer)

#### Estimation of the potential of TB-sensitive cash transfers to prevent catastrophic costs

To estimate the potential of cash transfers provided with a TB-sensitive approach to prevent catastrophic costs, we considered that cash transfers would be targeted to poor households at high risk of developing active TB disease, to increase their pre-illness income and protect them from poverty. For any beneficiary households that later developed active TB disease, their cash transfer-increased household income would make them more resilient to the burden of TB-related costs incurred pre- and during-treatment. Thus, we added the value of cash transfers to pre-illness annual household income and then recalculated countries’ TB-related cost burden (Box 2, Eq 2).

#### Estimation of TB-specific and TB-sensitive cash transfer needed to prevent catastrophic costs

To estimate the total value of cash transfer that would be needed by poor TB-affected households to prevent catastrophic costs with a TB-specific versus a TB-sensitive approach, we considered that for each approach, an additional cash transfer would be provided to targeted households to achieve this objective. Thus, we first estimated countries’ household-level additional cash transfer needed to prevent catastrophic costs by rearranging Eqs 1 and 2, fixing countries’ TB-related cost burden at 20%, and considering an unknown value of additional cash transfer (Box 2, Eqs 3ii and 4ii). Then we estimated countries’ household-level total cash transfer needed to prevent catastrophic costs by adding the value of original cash transfer to our estimated value of additional cash transfer needed to prevent catastrophic costs.

#### Estimation of TB-specific and TB-sensitive cash transfer budget needed to prevent catastrophic costs

To estimate the country-level budget that countries would need to prevent catastrophic costs for all poor households targeted with a TB-specific versus a TB-sensitive approach, we considered that for each approach a value of cash transfer sufficient to prevent catastrophic costs would be provided to all targeted households. Thus, we multiplied countries’ estimated TB-specific and TB-sensitive household-level total cash transfer needed to prevent catastrophic costs by the size of each approach’s target population, which for a TB-specific approach was all households with a confirmed TB diagnosis in the countries’ poorest population quintile, and for a TB-sensitive approach was households in poverty already targeted by countries’ established governmental poverty-reduction cash transfer programme.

### Sensitivity analysis

We tested the sensitivity of our results in Brazil, Colombia, Tanzania, and Mexico to imputation of missing DS TB-related cost components by repeating our analysis omitting rather than imputing the value of missing DS TB-related cost components [7]. We separately tested the sensitivity of our results across all countries included in the study to the use of 20% as the threshold for measuring countries’ TB-related cost burden as catastrophic. We did this by repeating our analyses instead using a 10% and 30% threshold.

## Results

**Fig 1:**
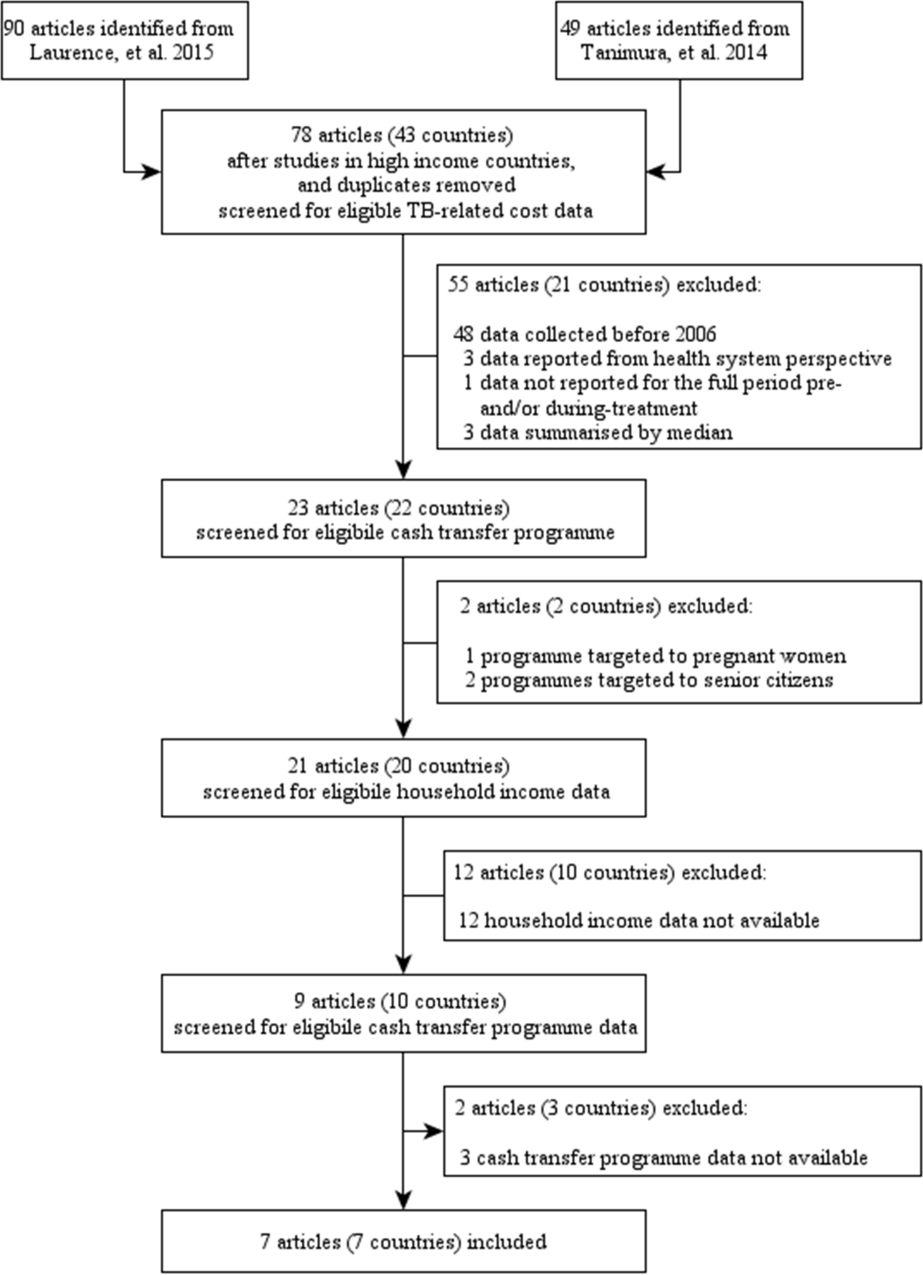
Flow chart of country eligibility and TB-related cost survey inclusion in the study. TB, tuberculosis.

Fig 1 is a flow chart of the review process for assessing the eligibility of countries for inclusion in this study. Argentina, Bangladesh, and South Africa had to be excluded after insufficient publically available background information was identified for eligible cash transfer programmes in these countries. Consequently, 7 countries were included in the data analysis.

### Summary of DS TB-related cost data

Conducted in Brazil, Colombia, Ecuador, Ghana, Mexico, Tanzania, and Yemen between 2006 and 2012, survey sample sizes ranged from 94 to 320 patients with active DS TB disease (Table 1). Surveys collected data on DS TB-related costs incurred pre- and during-treatment, except in Brazil [33], Colombia [62], and Tanzania [63], where they only collected data during-treatment (Table 1). Surveys collected both direct and indirect cost data, except in Mexico [64] where no data was collected characterising indirect costs (Table 1). In countries where data was collected, methods for estimating indirect costs varied in 2 ways: 1) reported time lost travelling and waiting to receive TB care was multiplied by patients’ reported income [33,65]; or 2) reported time lost travelling and waiting to receive TB care was multiplied by an estimate of national average income (gross national income per capita or official wage rate) [33,62,63,66]. In Ecuador [66], data was collected on additional costs described in the publication as referring to “loans, paying for additional help and other impacts throughout the course of TB illness.” The ambiguity of this cost category meant that it could not be classified as either direct or indirect costs and was thus reported as its own subcategory. Reported mean DS TB-related total costs for the complete TB illness ranged from $387 to $2,382 (Table 1). After imputing missing TB-related cost components in Brazil, Colombia, Mexico and Tanzania, estimated mean DS TB-related total costs ranged from $774 (95% CI: $618–$930) to $5,954 (95% CI: $4,997–$6,911), Table 1.

**Table 1:** Summary of TB-related cost surveys included in the study.

Cost survey			Reported TB-related costs					Estimated TB-related costs
Country	Year	Number of participants	Treatment phase	Direct 2013 PPP$	Indirect 2013 PPP$	Additional 2013 PPP$	Total 2013 PPP$	Total 2013 PPP$ (95% CIs) *
**DS TB**			** **	** **	** **	** **	** **	
Brazil§ [33]	2010	218	During-	182	205		387	774 (618–930) †
Ecuador [66]	2007	104	Pre- and during-	846	860	620	2,326	2,326 (1,834–2,818)
Yemen§ [34]	2008/09	320	Pre- and during-	631	253		885	885 (778–992)
Tanzania [63]	2012	94	During-	506	330		836	1,672 (1,300–2,044) †
Ghana [65]	2009	135	Pre- and during-	326	883		1,208	1,208 (984–1,432)
Colombia [62]	2010	150	During-				707	1,414 (1,165–1,663) †
Mexico [64]	2007/08	180	Pre- and during-	2,382			2,382	5,954 (4,997–6,911) ‡
**DR TB**								
Ecuador [66]	2007	14	Pre- and during-	2,345	4,560	9,762	16,667	16,667 (7,063–26,271)

Abbreviations: CI, confidence interval; DR, drug-resistant; DS, drug-susceptible; PPP, Purchasing power parity; TB, tuberculosis.

*According to Tanimura et al., estimated total costs in all countries had a standard deviation with a ratio of 1.1 to their value [7]. The probability distribution of TB-related costs was assumed to be normal. This was justified because our analysis was at the national level and we used mean values.

†According to Tanimura et al., reported during-treatment costs were assumed to only represent 50% of total TB-related costs [7].

‡According to Tanimura et al., reported direct costs pre- and during-treatment were assumed to only represent 40% of total TB-related costs [7].

§TB-related costs were extracted as an unweighted mean overall estimate calculated across patient subgroups.

### Summary of DR TB-related cost data

Conducted in Ecuador in 2007, the survey sample size was 14 patients with active multidrug-resistant TB disease, Table 1. The survey reported mean DR TB-related costs incurred pre- and during-treatment (Table 1). The survey collected both direct and indirect cost data (Table 1). Cost data was also collected on additional costs. This category of costs was reported as its own subcategory. Indirect costs were estimated by multiplying reported time lost travelling and waiting to receive TB care by the estimated hourly wage in Ecuador. Mean DR TB-related total costs were $16,667 (95% CI: $7,063–$26,271), Table 1.

### Summary of cash transfer data

All extracted cash transfer data refer to programmes’ status in 2013. Mean cumulative annual cash transfers were greatest in Brazil, Colombia, Ecuador, Mexico, and Yemen varying between $823 (range: $239–$1,084) and $1,091 (range: $1,091–$1,091); and lowest in Ghana and Tanzania where they were $217 (range: $150–$299) and $451 (range: $349–$655), respectively (Table 2). Across countries, cash transfers ranged from 7.7% (95% CI: 7.6%–7.9%) to 43% (95% CI: 42%–44%) of annual household income. In Colombia, Ecuador, Ghana, Mexico, and Tanzania they varied between 13% (95% CI: 11%–17%) and 59% (95% CI: 50%–72%) of DS TB-related costs, and in Brazil and Yemen, respectively, they were 104% (95% CI: 93%–119%) and 106% (95% CI: 88%–133%) of DS-TB-related costs (Table 2). In Ecuador, cash transfers represented 7.3% (95% CI: 4.2%–15%) of DR TB-related costs (Table 2). A summary of cash transfer data sources and additional extracted data is provided in S1 Table.

**Table 2:** Summary of poverty-reduction cash transfer programmes included in the study and TB-specific versus TB-sensitive target populations.

Poverty-reduction cash transfer programme				Current poverty-reduction cash transfer % of		Target population, in households	
Country	Name	Components	Current cash transfer 2013 PPP$ (Range)	Household income† (95% CIs) §	TB-related costs (95% CIs) §	TB-specific (% of population)	TB-sensitive (% of population)
**DS TB**		** **			** **	** **	
Brazil	Programa Bolsa Familia [36,37]	Flat benefit to extremely poor families; and variable benefits to poor families to support child health, child/adolescent education, and pregnant women’s health	823 (239–1,084)	15 (15–16)	106 (88–133)	34,800 (0.06)	14,000,000 (25)
Ecuador	Bono de Desarollo Humano [38]	Flat benefit to poor families to support child health and education	1,091 (1,091–1,091)	13 (12–13)	47 (39–59)	3,520 (0.09)	450,000 (12)
Yemen	Social Welfare Fund [39]	Flat benefit to poor families; and variable benefit to poor families for household size	923 (615–1,026) ᶲ	43 (42–44)	104 (93–119)	4,800 (0.17)	1,500,000 (35)
Tanzania	Productive Social Safety Net [40–42]	Flat benefit for poor families; and variable benefits to poor families to support child health and education and pregnant women’s health	217 (150–299)	7.7 (7.6–7.9)	13 (11–17) ‡	67,600 (0.72)	150,000 (2)
Ghana	Livelihood Empowerment Against Poverty [43]	Variable benefit to poor families for orphans and vulnerable children, disabled and those over 65	451 (349–655)	25 (24–26)	37 (32–46)	17,600 (0.32)	70,000 (1)
Colombia	Mas Familias en Accion [44,45]	Variable benefits to poor families to support child health and child/adolescent education	837 (191–1,777)	38 (37–39)	59 (50–72)	6,000 (0.06)	26,000,000 (25)
Mexico	Oportunidades* [46,47]	Variable benefits to poor families to support family health, child/adolescent education, family nutrition	940 (246–2,063)	20 (19–20)	16 (14–19)	10,000 (0.04)	6,600,000 (27)
**DR TB**		** **	** **	** **	** **	** **	
Ecuador	Bono de Desarollo Humano [38]	Flat benefit to poor families to support child health and education	1,091 (1,091–1,091)	13 (12–13)	7.3 (4.2–15)	300 (0.01)	450,000 (12)

Apart from countries’ alternative target populations, all data are mean estimates.

Abbreviations: CI, confidence interval, DR, drug-resistant; DS, drug-susceptible; PPP, purchasing power parity; TB, tuberculosis.

*Formerly PROGRESA.

†Household income refers to average pre-illness annual household income in the poorest population quintile.

‡Household income was extracted as household expenditure in the poorest population quintile.

§To estimate 95% confidence intervals, all mean TB-related costs were assumed to have a standard deviation with a ratio of 1.1 to their value, all mean household incomes were assumed to have a standard deviation with a ratio of 0.8 to their value, and all mean cash transfers were assumed to have a standard deviation equal to a quarter of maximum minus minimum cash transfers. Probability distributions for all 3 input parameters were assumed to be normal. This was justified because our analysis was at the national level and we used mean values.

ᶲBecause of changes in cash transfer programme administration in the study year, reported mean cash transfers were higher than the maximum value of cash transfers able to be received by beneficiary households in 2013 [39]. We assumed that mean cash transfers were approximately equivalent to the value that would be received by an average household in the country's poorest population quintile based on household size.

### Summary of household income data

Conducted between 2005 and 2011, survey sample sizes ranged from 8,687 to 55,970 households [55–61]. Surveys reported mean household income, except in Tanzania where mean household expenditure was reported. Estimated mean annual household income in countries’ poorest population quintiles was highest in Brazil, Ecuador, and Mexico varying between $4,755 and $8,692, and lowest in Colombia, Ghana, Tanzania, and Yemen varying between $1,617 and $2,812. A summary of annual household income data sources and extracted data is provided in S1 Table.

### Summary of target population data

For DS TB, the size of countries’ estimated TB-specific target population, which was equivalent to 40% of countries’ TB burden, ranged from 3,520 to 67,600 households, and the size of countries’ estimated TB-sensitive target population, which was equivalent to the number of households in poverty already targeted by countries’ established poverty-reduction cash transfer programme, ranged from 70,000 to 26 million households (Table 2). For DR TB, the size of Ecuador’s estimated TB-specific target population was 300 households, and the size of its estimated TB-sensitive target population was 450,000 households (Table 2).

### Summary of DS TB-related cost burden before cash transfers

**Fig 2:**
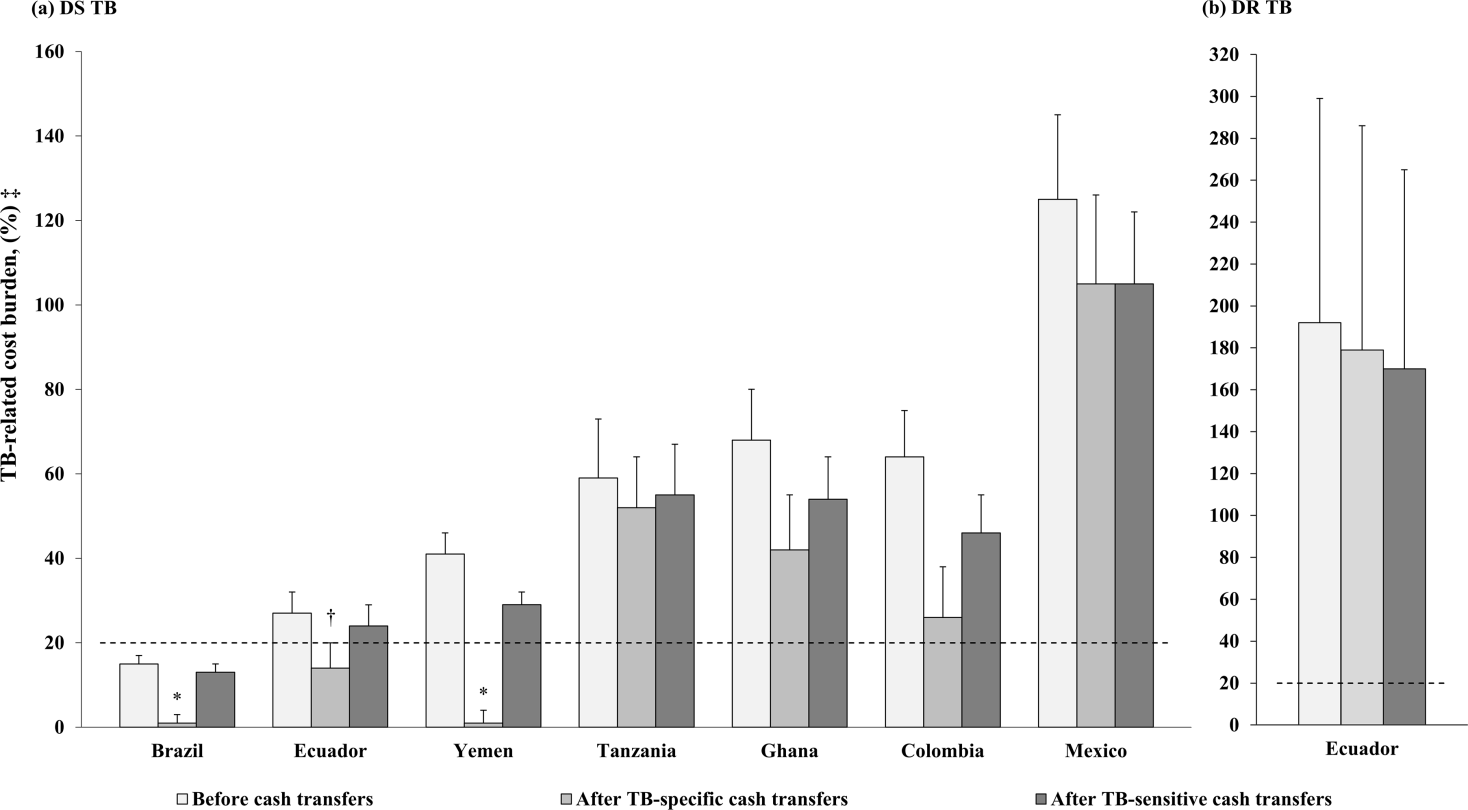
Summary of countries’ household-level TB-related cost burden before, and after cash transfers. The “Before cash transfers” bar represents countries’ mean TB-related cost burden without cash transfer data. The “After TB-specific cash transfers” bar represents countries’ mean TB-related cost burden after cash transfers have been subtracted from TB-related costs. The “After TB-sensitive cash transfers” bar represents countries’ mean TB-related cost burden after cash transfers have been added to countries’ pre-illness household income. The dotted line guides whether countries’ mean TB-related cost burden is above or below 20%. Error bars represent 95% CIs calculated using the quantile method. The values used to build Fig 2 are provided in S2 Table. *For clarity, a mean TB-related cost burden of 0% after cash transfers is plotted as 0.9%. †Upper bound of 95% CI = 19.8. ‡To estimate 95% CIs, all mean TB-related costs were assumed to have a standard deviation with a ratio of 1.1 to their value [7], all mean household incomes were assumed to have a standard deviation with a ratio of 0.8 to their value [37,44], and all mean cash transfers were assumed to have a standard deviation equal to a quarter of maximum minus minimum cash transfers. Probability distributions for all 3 input parameters were assumed to be normal. This was justified because our analysis was at the national level and we used mean values. DR, drug-resistant; DS, drug-susceptible; TB, tuberculosis.

Before cash transfers, estimated DS TB-related cost burdens varied between 15% (95% CI: 12%–18%) and 125% (95% CI: 105%–145%) of annual household income, and were catastrophic in Colombia, Ecuador, Ghana, Mexico, Tanzania, and Yemen where they varied between 27% (95% CI: 21%–32%) and 125% (95% CI: 105%–145%) of annual household income (Fig 2).

### Summary of the potential of TB-specific cash transfers to prevent DS TB catastrophic costs, and the budget needed for this approach

**Fig 3:**
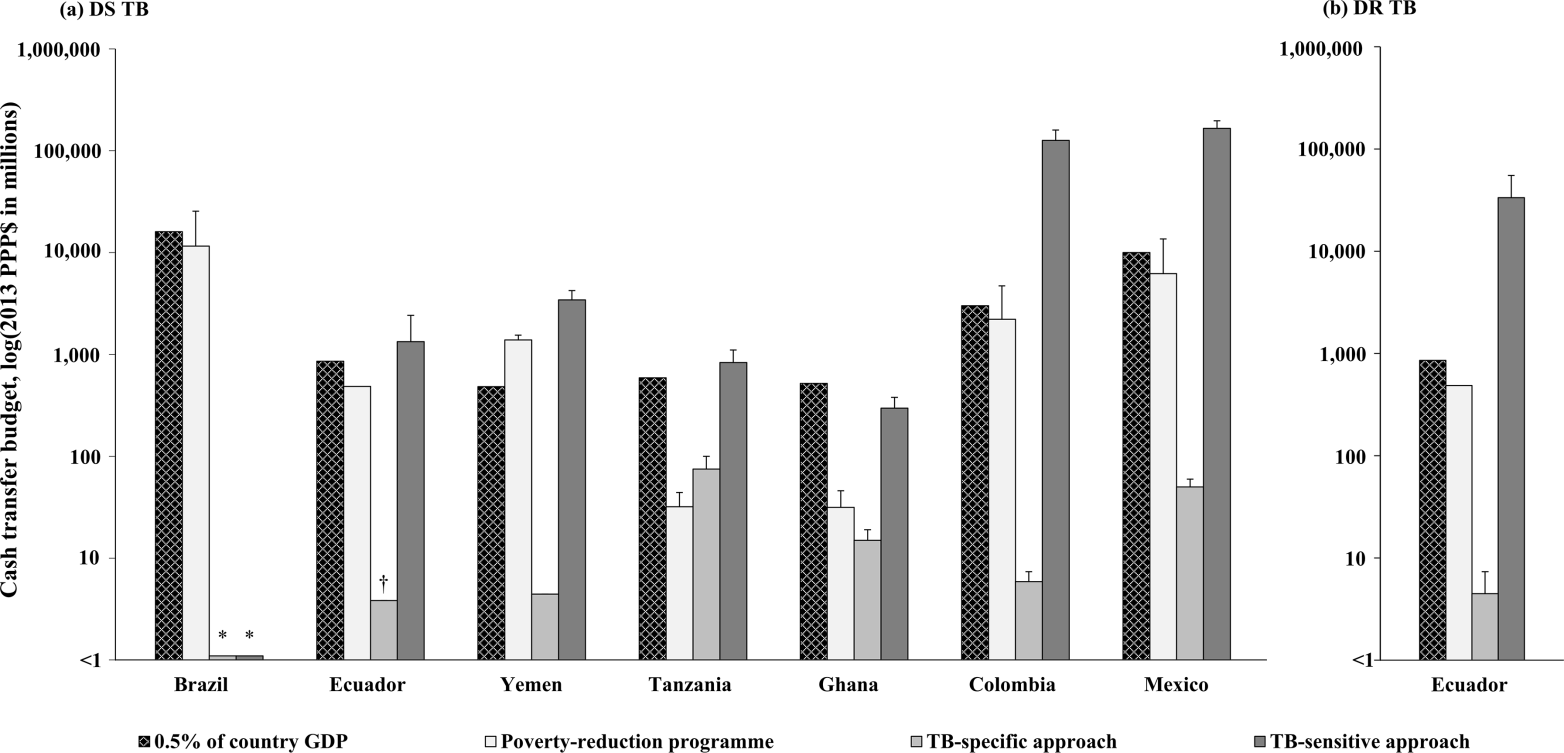
Summary of countries’ country-level cash transfer budget needed to prevent catastrophic costs. All data are expressed in millions on the log_10_ scale. We summarise 0.5% of countries’ GDP and their existing poverty-reduction cash transfer budget for comparison. The “0.5% of country GDP” bar represents the upper limit that governments in low- and middle-income countries spend on a poverty-reduction cash transfer programme [27]. The “poverty-reduction programme” bar represents countries’ actual poverty-reduction cash transfer programme budget. The “TB-specific approach” bar represents the mean budget that countries would need to prevent their TB-specific target population from incurring catastrophic costs. The “TB-sensitive approach” bar represents the mean budget that countries would need to prevent their TB-sensitive target population from incurring catastrophic costs. The values used to build Fig 3 are provided in S3 Table. *For clarity, a value of country-level cash transfer budget needed equal to $0 is plotted as $1.1. †Because data were highly skewed we reported median instead of mean. DR, drug-resistant; DS, drug-susceptible; GDP, gross domestic product; TB, tuberculosis.

If cash transfers were applied using a TB-specific approach to defray TB-related costs incurred by households with a confirmed DS TB diagnosis, then on average, they were sufficient to prevent catastrophic costs in Ecuador and Yemen, but insufficient to prevent them in either Colombia, Ghana, Mexico, or Tanzania (Fig 2). In Colombia, Ghana, Mexico, or Tanzania, the DS TB-related cost burden after TB-specific cash transfers varied between 26% (95% CI: 15%–38%) and 106% (95% CI: 86%–126%), and the estimated value of household-level additional TB-specific cash transfer needed to prevent DS TB catastrophic costs varied between $144 (95% CI: $0.0–$387) and $4,071 (95% CI: $3,122–$5,014), Table 3. In the 6 countries where TB-related costs were originally catastrophic, the estimated value of household-level total TB-specific cash transfer needed to prevent DS TB catastrophic costs varied between $850 (95% CI: $627–$1,079) and $5,011 (95% CI: $4,063–$5,952), Table 3. According to the size of countries’ TB-specific target populations, this value translated into a TB-specific country-level cash transfer budget needed to prevent DS TB catastrophic costs varying between $3.8 million (95% CI: $3.8 million–$3.8 million) and $75 million (95% CI: $50 million–$100 million), Fig 3.

**Table 3:** Summary of countries’ household-level additional and total cash transfer needed to prevent catastrophic costs.

	Additional cash transfer, 2013 PPP$ (95% CIs) *		Total cash transfer, 2013 PPP$ (95% CIs) *	
Country	TB-specific approach	TB-sensitive approach	TB-specific approach	TB-sensitive approach
**DS TB**	** **	** **		
Brazil	0.0 (0.0–0.0)	0.0 (0.0–0.0)	0.0 (0.0–0.0)	0.0 (0.0–0.0)
Ecuador	0.0 (0.0–0.0) †	1,842 (0.0–4,281)	1,091 (1,091–1,091) †	2,971 (1,091–5,372)
Yemen	0.0 (0.0–0.0)	1,360 (821–1,897)	923 (920–926)	2,282 (1,743–2,819)
Tanzania	880 (510–1,243)	5,322 (3,473–7,139)	1,111 (741–1,474)	5,553 (3,707–7,370)
Ghana	399 (176–628)	3,800 (2,683–4,945)	850 (627–1,079)	4,251 (3,134–5,396)
Colombia	144 (0.0–387)	4,023 (2,764–5,281)	981 (830–1,223)	4,860 (3,600–6,117)
Mexico	4,071 (3,122–5,014)	24,115 (19,374–28,817)	5,011 (4,063–5,952)	25,055 (20,316–29,761)
**DR TB**	** **	** **		
Ecuador	13,782 (4,274–23,376)	73,275 (25,736–121,246)	14,877 (5,365–24,467)	74,375 (26,827–122,337)

All data are average estimates. For interpretability, negative estimates and confidence intervals are reported as 0.

Abbreviations: CI, confidence interval; DR, drug-resistant; DS, drug-susceptible; PPP, purchasing power parity; TB, tuberculosis.

*To estimate 95% confidence intervals, all mean TB-related costs were assumed to have a standard deviation with a ratio of 1.1 to their value [7], all mean household incomes were assumed to have a standard deviation with a ratio of 0.8 to their value [37,44], and all mean cash transfers were assumed to have a standard deviation equal to a quarter of maximum cash transfers minus minimum cash transfers. Probability distributions for all 3 input parameters were assumed to be normal. This was justified because our analysis was at the national level and we used mean values.

†Because data were highly skewed we reported median instead of mean.

### Summary of the potential of TB-sensitive cash transfers to prevent DS TB catastrophic costs, and the budget needed for this approach

If cash transfers were provided using a TB-sensitive approach to increase pre-illness income of poor households with high risk of developing active TB disease, then on average, for those that later develop active DS TB disease, this would not be sufficient to prevent them from incurring catastrophic costs in any of the 6 countries where DS TB-related costs were originally catastrophic (Fig 2). In these 6 countries, the DS TB-related cost burden after TB-sensitive cash transfers varied between 24% (95% CI: 19%–29%) and 105% (95% CI: 88%–121%), and the estimated value of household-level additional TB-sensitive cash transfer needed to prevent DS TB catastrophic costs varied between $1,360 (95% CI: $821–$1,897) and $24,115 (95% CI: $19,374–$28,817), Table 3. The estimated value of household-level total TB-sensitive cash transfer needed to prevent DS TB catastrophic costs varied between $2,282 (95% CI: $1,743–$2,819) and $25,055 (95% CI: $20,316–$29,761), Table 3. According to the size of countries’ TB-sensitive target populations, this value translated into a TB-sensitive country-level cash transfer budget needed to prevent DS TB catastrophic costs varying between $298 million (95% CI: $219 million–$378 million) and $165,367 million (95% CI: $134,085 million–$196,425 million), Fig 3.

### Summary of the potential of TB-specific versus TB-sensitive cash transfers to prevent DR TB catastrophic costs, and the budget needed for each approach

In Ecuador, the DR TB-related cost burden before cash transfers was 192% (95% CI: 86%–299%), Fig 2. Here, cash transfers provided with either a TB-specific or a TB-sensitive approach were, on average, insufficient to prevent DR TB catastrophic costs (Fig 2). The estimated value of TB-specific versus TB-sensitive additional cash transfer needed to achieve this objective was $13,782 (95% CI: $4,274–$23,376) versus $73,275 (95% CI: $25,736–$121,246); and the estimated value of household-level total TB-specific versus TB-sensitive cash transfers needed was $14,877 (95% CI: $5,365–$24,467) versus $74,375 (95% CI: $26,827–$122,337), Table 3. According to the size of Ecuador’s DR TB-specific and DR TB-sensitive target population, this value translated into a country-level cash transfer budget needed to prevent DR TB catastrophic costs of $4.5 million (95% CI: $1.6 million–$7.3 million) with a TB-specific approach versus $33,469 million (95% CI: $12,072 million–$55,052 million) with a TB-sensitive approach (Fig 3).

### Sensitivity analysis without imputing data

Before cash transfers, the TB-related cost burden remained catastrophic in the same countries as when missing TB-related cost components were imputed, and the only difference after cash transfers was that TB-specific cash transfers prevented catastrophic costs in Colombia (S4 Table). Across countries, TB-specific cash transfers remained more affordable at preventing catastrophic costs compared to TB-sensitive cash transfers both at the household and country level (S5 Table).

### Sensitivity analysis with 10% threshold

Before cash transfers, in addition to Colombia, Ecuador, Ghana, Mexico, Tanzania, and Yemen, the DS TB-related cost burden was also catastrophic in Brazil. In Ecuador, the DR TB-related cost burden before cash transfers remained catastrophic. Across countries, TB-specific cash transfers remained more affordable than TB-sensitive cash transfers for preventing DS and DR TB catastrophic costs both at the household and country level (S6 Table).

### Sensitivity analysis with 30% threshold

Before cash transfers, the DS TB-related cost burden remained catastrophic in Colombia, Ghana, Mexico, Tanzania, and Yemen, but ceased to be catastrophic in Ecuador. In Ecuador, the DR TB-related cost burden before cash transfers remained catastrophic. Across countries, TB-specific cash transfers remained more affordable than TB-sensitive cash transfers for preventing DS and DR TB catastrophic costs both at the household and country level (S7 Table).

## Discussion

In the 7 countries that met our inclusion criteria, our analysis of national average data suggests that DS TB-related costs would be catastrophic for the average poor TB-affected household in most low- and middle-income countries. This is concerning and concordant with the limited evidence that is already available [7]. If cash transfers were provided with a TB-specific approach to defray TB-related costs of poor households with a confirmed DS TB diagnosis, then in some low- and middle-income countries, they would likely be sufficient to prevent the average household incurring DS TB catastrophic costs. Alternatively, if the same value of cash transfers were provided with a TB-sensitive approach to increase the income and strengthen the economic resilience of poor households at high risk of developing active TB disease, then across low- and middle-income countries, they would likely be insufficient to prevent the average household that later developed active DS TB disease incurring DS TB catastrophic costs. In countries where neither TB-specific nor TB-sensitive cash transfers would be sufficient to prevent DS TB catastrophic costs, the average value of household-level additional cash transfer needed to achieve this objective would be much lower using a TB-specific approach compared to a TB-sensitive approach. Further, by only targeting poor households with a confirmed TB diagnosis, a TB-specific approach would, on average, require a much smaller country-level budget than using a TB-sensitive approach to target much larger numbers of poor households at high risk of developing active TB disease.

Although DR TB is rare, it is associated with extreme TB-related costs [7]. Neither TB-specific nor TB-sensitive cash transfers would be sufficient to prevent DR TB catastrophic costs for the average poor DR TB-affected household. The value of household-level additional cash transfer needed to achieve this objective would be very high. Because few poor households are affected by DR TB, countries’ county-level cash transfer budget needed to prevent DR TB catastrophic costs would, on average, be much lower using a TB-specific approach compared to a TB-sensitive approach. Given that so few households are affected by DR TB, it may not be rational for TB-sensitive cash transfer programmes to aim to increase households’ annual income sufficiently to make all poor households resilient to the rare and extreme costs associated with DR TB.

To our knowledge, our study is the first to compare the potential of cash transfers provided with a TB-specific versus a TB-sensitive approach to prevent catastrophic costs. The contrasting effects of defraying TB-related costs using a TB-specific approach versus increasing households’ pre-illness income using a TB-sensitive approach has important and novel implications for protecting TB-affected households from catastrophic costs. We believe our study is also the first to compare the country-level cash transfer budget that would be needed to prevent catastrophic costs for poor TB-affected households using a TB-specific versus a TB-sensitive approach. We show that by being more effective and aiming to reach fewer households, a TB-specific approach would cost less than a TB-sensitive approach. It is important to emphasize that these findings are only valid when preventing catastrophic costs is the only outcome of interest. Cash transfers provided to poor households with a TB-sensitive approach might have far-reaching effects on wellbeing, health promotion, and disease prevention, and further evaluation is needed to study the costs versus benefits of each approach [24,67]. Nevertheless, the End TB Strategy prioritises ensuring that 0 TB-affected households experience catastrophic costs [17]. For achieving this specific milestone, the implications of our study are clear: cash transfers provided with a TB-specific approach are likely to achieve this goal more affordably than if they were provided with a TB-sensitive approach.

Our study adds to limited evidence informing the best targeting strategy for cash transfers aimed at enhancing TB care and prevention [24]. At the country-level, showing that in Latin America and Central Asia a TB-sensitive approach might reach between 12% and 35% of countries’ population, whereas in some parts of sub-Saharan Africa it might only reach between 1% and 2% of countries population, this study supports speculation that the potential of countries to provide cash transfers with a TB-sensitive approach might follow an inverse care law [68], whereby poorer countries with higher TB burdens have less well established poverty-reduction cash transfer programmes [24]. Showing also that approximately 40% of TB-affected households might be in the poorest population quintile, this study highlights the need to consider how cash transfers might be targeted to households that incur catastrophic costs but are outside of this population category [50,51]. With a TB-specific approach, it would be relatively easy to modify programmes’ target population to include more TB-affected households, whilst with a TB-sensitive approach, it might be harder to modify the target population of existing poverty-reduction programmes, which are usually well-established parts of national social protection systems [35].

This study has several limitations, and conclusions should be drawn cautiously. Insufficient data forced us to estimate the potential of TB-specific versus TB-sensitive cash transfers to prevent catastrophic costs using the value of cash transfers offered by existing governmental poverty-reduction cash transfer programmes. Whilst the only solution, it will have nonetheless under- or overestimated the potential of TB-specific cash transfers depending on how their actual value compares to governmental poverty-reduction cash transfers. Our inputs were all associated with some uncertainty, especially TB-related costs, which were mostly extracted from small subnational cost surveys [33,34,62–66]. We attempted to account for this using a multiway analysis that allowed inputs to vary by their simulated sampling distributions. Inconsistent reporting of standard deviations for mean TB-related costs, household income, and cash transfers forced us to make assumptions about the amount of variance around extracted values and to generalise these across countries. Whilst we ensured that our estimates of variance were as accurate as possible by drawing from relevant literature [7,37,44], this approach will have ignored any country-specific skewness or kurtosis in input parameters. Inconsistent reporting of TB-related costs disaggregated by income quintile meant that we had to assume that estimated TB-related costs were representative of those incurred by affected households in countries’ poorest population quintile. Because poorer households usually incur lower TB-related costs compared to less poor households, this is likely to have underestimated the potential of cash transfers to prevent catastrophic costs, and overestimated the country-level budget needed to achieve this [14]. Whilst our analysis should provide an accurate estimate of the effect of cash transfers on countries’ mean TB-related cost burden, because sample distributions of TB-related costs are known to be positively skewed [7], the aggregate-level nature of our study means that our results are unlikely to be representative of the majority of TB-affected households. Two sources of error in this study were imputation of missing TB-related cost components in Brazil [33], Colombia [62], Tanzania [63], and Mexico [64], and measuring catastrophic costs using a threshold TB-related cost burden that still hasn’t been assessed to determine its clinical or financial relevance for TB-affected households in any of the countries included in the study. Sensitivity analysis showed that the potential of cash transfers to prevent catastrophic costs was robust to these sources of error, but the precise estimates of countries’ household-level additional and total cash transfer and country-level cash transfer budget needed to prevent catastrophic costs were dependent on them. Therefore, whilst a TB-specific approach was consistently more effective and affordable for preventing catastrophic costs compared to a TB-sensitive approach in all our analyses, further research is needed to precisely estimate the cost of each of these approaches.

Our study is consistent with, and adds to, individual level evidence supporting the potential of TB-specific cash transfers to prevent catastrophic costs for poor TB-affected households in Peru [15]. Whilst our study questions the ability of TB-sensitive cash transfers to prevent households from engaging in damaging coping strategies, it supports individual evidence from Latin America for their ability to improve households’ coping capacity in response to severe livelihood risks [21]. By focussing on preventing catastrophic costs in low- and middle-income countries, it adds another dimension to the 2015 Cochrane review of the use of cash transfers in TB control, which did not find evidence on this outcome and mostly examined the use of cash transfers in the United States [69]. For future research, the validity of our results should be tested using individual-level primary data from future TB-related cost surveys, secondary data that includes information on households’ TB exposure, income and social protection status [70], and/or experimental data from interventions like the ongoing CRESIPT trial in Peru [25,26]. This work should also look to explore the effect of TB-specific and TB-sensitive cash transfers on other proxy measures of catastrophic costs like household dissaving (e.g., taking out a loan and/or selling household items) [15,16]. For a more complete understanding of the impact of providing cash transfers with a TB-specific versus a TB-sensitive approach, future research should also aim to incorporate this study’s data into an epidemiological model that accounts for their respective effects on TB-related catastrophic costs, and the additional effects of a TB-sensitive approach on individuals’ risk of TB infection and progression by addressing poverty-related risk factors (e.g., poor living conditions and undernutrition) [71,72].

In addition to studying the potential of cash transfers to prevent TB-related catastrophic costs, future research should also prioritise investigating the effect of other forms of social protection on this outcome. For example, in Mexico [64], food assistance might effectively defray households’ high direct nonmedical food costs [73], and in Ghana [65], facilitating patients’ access to sickness benefits and their prompt reintegration into the labour market might help to avoid high indirect costs [74]. Obviously, social protection should not be implemented in isolation of other healthcare initiatives to reduce costs. Further research should also aim to evaluate the complementary effects of social protection and efforts to reduce out of pocket direct medical costs. For example, combining social protection with further investments to maximize ambulatory community-based care might be especially effective for preventing catastrophic costs in Ecuador, where patients incur high direct medical costs for hospitalisation [66,75,76]. Multidisciplinary research platforms like the Health and Social Protection Action Research & Knowledge Sharing (SPARKS) Network will be key for facilitating this sort of research [77].

Our analysis compares cash transfers provided with a TB-specific versus TB-sensitive approach. Interestingly, it has been proposed that one efficient and cost-effective strategy might be to integrate both TB-specific and TB-sensitive approaches into a so-called “TB-inclusive” approach [24]. Results from our study demonstrating the greater potential of TB-specific cash transfers to prevent catastrophic costs, and the existing coverage of TB-sensitive cash transfer programmes, may support this integrated approach. For a TB-inclusive approach, existing poverty-reduction programmes could be adapted to include an additional variable TB-specific benefit, which beneficiary households would be eligible to receive upon receipt of a confirmed TB diagnosis. To finance such an approach, stakeholders from across TB control, development, and finance sectors could coordinate to determine how much each would be willing to contribute given their separate objectives [78,79]. From a TB prevention perspective, such an investment would be expected to reduce delays for TB diagnosis [10], reduce the risk of adverse treatment outcomes [14], and thus potentially contribute to reduced national TB incidence [80]. From the perspective of social development, reduced national TB incidence would be expected to enable previously vulnerable households to invest more in human capital, increase their labour productivity, and thus contribute to long-term sustainable economic growth [81]. Because households affected by human immunodeficiency virus (HIV), mental health issues, diabetes, and other noncommunicable diseases are also known to incur high direct and indirect costs [82,83], any efforts to prevent catastrophic costs for TB-affected households should aim to collaborate with these other areas of public health. Whichever approach for providing cash transfers to prevent TB-related catastrophic costs is chosen, it will be key to ensure that it is not implemented in isolation from universal health coverage initiatives including more decentralised and patient-friendly TB service delivery. Social protection initiatives and universal health coverage initiatives should be developed and implemented hand-in-hand [19].

Our finding that neither a TB-specific nor a TB-sensitive approach might be sufficient to prevent DR TB catastrophic costs highlights the urgent need for considerable investments in social protection and universal health coverage initiatives targeted to households affected by this disease. Globally, only 20% of people with DR TB are estimated to begin treatment, and only 52% of those that start treatment are estimated to successfully complete it [1]. Therefore, DR TB-affected households should constitute a “special case” for future investments to prevent catastrophic costs.

Reviewing and analysing the literature on TB-related costs and poverty-reduction cash transfer programmes in low- and middle-income countries, our study compares the potential of cash transfers provided with a TB-specific versus a TB-sensitive approach to prevent catastrophic costs for poor TB-affected households. Our findings suggest that providing cash transfers with a TB-specific approach to defray TB-related costs of households with a confirmed TB diagnosis will be more effective and affordable for achieving this objective compared to a TB-sensitive approach that increases the income and strengthens the economic resilience of households at high risk of developing active TB disease. Our findings also highlight an urgent need for investments to prevent catastrophic costs for households having to confront the severe medical, social, and economic challenges caused by DR TB.

## Supporting information

S1 CHEERS checklist (DOCX)

S1 Text Prospective analysis plan. (DOCX)

S1 Table Summary of cash transfer and household income data sources. PPP, purchasing power parity.
(DOCX)

S2 Table Summary of countries’ household-level TB-related cost burden before, and after cash transfers. The “Before cash transfers” column represents countries’ mean TB-related cost burden without cash transfer data. The “After TB-specific cash transfers” column represents countries’ mean TB-related cost burden after cash transfers have been subtracted from TB-related costs. The “After TB-sensitive cash transfers” column represents countries’ mean TB-related cost burden after cash transfers have been added to countries’ pre-illness household income. CI, confidence interval; DR, drug-resistant; DS, drug-susceptible; TB, tuberculosis.
(DOCX)

S3 Table Summary of countries’ country-level cash transfer budget needed to prevent catastrophic costs. The “0.5% of country GDP” column represents the upper limit that governments in low- and middle-income countries spend on a poverty-reduction cash transfer programme [27]. The “poverty-reduction programme” column represents countries’ actual poverty-reduction cash transfer programme budget. The “TB-specific approach” column represents the mean budget that countries would need to prevent their TB-specific target population from incurring catastrophic costs. The “TB-sensitive approach” column represents the mean budget that countries’ would need to prevent their TB-sensitive target population from incurring catastrophic costs. CI, confidence interval; DR, drug-resistant; DS, drug-susceptible; GDP, gross domestic product; TB, tuberculosis.
(DOCX)

S4 Table Summary of countries’ household-level TB-related cost burden before and after cash transfers without imputation of missing costs components. The “Before cash transfers” column represents countries’ mean TB-related cost burden without cash transfer data. The “After TB-specific cash transfers” column represents countries’ mean TB-related cost burden after cash transfers have been subtracted from TB-related costs. The “After TB-sensitive cash transfers” column represents countries’ mean TB-related cost burden after cash transfers have been added to countries’ pre-illness household income. CI, confidence interval; DS, drug-susceptible; TB, tuberculosis.
(DOCX)

S5 Table Summary of countries’ household-level additional and total cash transfer, and country-level cash transfer budget needed to prevent catastrophic costs without imputation of missing costs components. The “additional cash transfer” column represents the additional value of cash transfer that countries’ average TB-affected household would need to prevent catastrophic costs using a TB-specific versus a TB-sensitive approach. The “total cash transfer” column represents the total value that countries’ average TB-affected household would need to prevent catastrophic costs using a TB-specific versus a TB-sensitive approach. The “cash transfer budget, in millions” column represents the mean budget that countries would need to prevent catastrophic costs for its TB-specific versus TB-sensitive target populations. CI, confidence interval; DS, drug-susceptible; PPP, purchasing power parity; TB, tuberculosis.
(DOCX)

S6 Table Summary of countries’ household-level additional and total cash transfer, and country-level cash transfer budget needed to prevent catastrophic costs using a 10% threshold TB-related cost burden for measuring catastrophic costs. The “additional cash transfer” column represents the additional value of cash transfer that countries’ average TB-affected household would need to prevent catastrophic costs using a TB-specific versus a TB-sensitive approach. The “total cash transfer” column represents the total value that countries’ average TB-affected household would need to prevent catastrophic costs using a TB-specific versus a TB-sensitive approach. The “cash transfer budget, in millions” column represents the mean budget that countries would need to prevent catastrophic costs for their TB-specific versus TB-sensitive target populations. CI, confidence interval; DR, drug-resistant; DS, drug-susceptible; PPP, purchasing power parity; TB, tuberculosis.
(DOCX)

S7 Table Summary of countries’ household-level additional and total cash transfer, and country-level cash transfer budget needed to prevent catastrophic costs using a 30% threshold TB-related cost burden for measuring catastrophic costs. The “additional cash transfer” column represents the additional value of cash transfer that countries’ average TB-affected household would need to prevent catastrophic costs using a TB-specific versus a TB-sensitive approach. The “total cash transfer” column represents the total value that countries’ average TB-affected household would need to prevent catastrophic costs using a TB-specific versus a TB-sensitive approach. The “cash transfer budget, in millions” column represents the mean budget that countries’ would need to prevent catastrophic costs for their TB-specific versus TB-sensitive target populations. CI, confidence interval; DR, drug-resistant; DS, drug-susceptible; PPP, purchasing power parity; TB, tuberculosis.
(DOCX)

## Abbreviations

CHEERS: Consolidated Health Economic Evaluation Reporting Standards
CRESIPT: Community Randomized Evaluation of a Socioeconomic Intervention to Prevent TB
DR: drug-resistant
DS: drug-susceptible
HIV: human immunodeficiency virus
IHSN: International Household Survey Network
SPARKS: Social Protection Action Research & Knowledge Sharing
TB: tuberculosis
$: international dollars
