# Supplementary material for: Comparison of two cash transfer strategies to prevent catastrophic costs for poor tuberculosis-affected households in low- and middle-income countries: An economic modelling study
Source: PLoS Med. 2017 Nov 7;14(11):e1002418. doi: 10.1371/journal.pmed.1002418 (PMC5675360; doi:10.1371/journal.pmed.1002418)
Supplement: S1 CHEERS checklist — (DOCX) [file pmed.1002418.s001.docx]

| Section/item | Item No | Recommendation | Quoted text addressing recommendation |
| --- | --- | --- | --- |
| **Title and abstract** |  |  |  |
| Title | 1 | Identify the study as an economic evaluation or use more specific terms such as “cost-effectiveness analysis”, and describe the interventions compared. | Title: "Comparison of two cash transfer strategies to prevent catastrophic costs for poor tuberculosis-affected households in low- and middle-income countries: An economic modelling study" |
| Abstract | 2 | Provide a structured summary of objectives, perspective, setting, methods (including study design and inputs), results (including base case and uncertainty analyses), and conclusions. | Abstract: "Background. Illness-related costs for patients with tuberculosis (TB) ≥20% of pre-illness annual household income predict adverse treatment outcomes and have been termed “catastrophic”. Social protection initiatives, including cash transfers, are endorsed to help prevent catastrophic costs. With this aim, cash transfers may either be provided to defray TB-related costs of households with a confirmed TB diagnosis (termed a “TB-specific” approach); or to increase income of households with high TB risk to strengthen their economic resilience (termed a “TB-sensitive” approach). The impact of cash transfers provided with each of these approaches might vary. We undertook an economic modelling study from the patient perspective to compare the potential of these two cash transfer approaches to prevent catastrophic costs.  Methods and Findings. Inputs in the seven low- and middle-income countries Brazil, Colombia, Ecuador, Ghana, Mexico, Tanzania and Yemen were retrieved by literature review, and included countries' mean patient TB-related costs, mean household income, mean cash transfers, and estimated TB-specific and TB-sensitive target populations. Analyses were completed for drug-susceptible (DS) TB-related costs in all 7/7 countries, and additionally for drug-resistant (DR) TB-related costs in the 1/7 countries with available data. All cost data were reported in 2013 international dollars ($). The target population for TB-specific cash transfers was poor households with a confirmed TB diagnosis, and for TB-sensitive cash transfers was poor households already targeted by countries’ established poverty-reduction cash transfer programme. Cash transfers offered in countries, unrelated to TB, ranged from $217 to $1,091/year/household. Before cash transfers, DS TB-related costs were catastrophic in 6/7 countries. If cash transfers were provided with a TB-specific approach, alone they would be insufficient to prevent DS TB catastrophic costs in 4/6 countries, and when increased enough to prevent DS TB catastrophic costs would require a budget between $4 million (95%CI: $4-$4 million) and $50 million (95%CI: $41-$60 million) per country. If instead cash transfers were provided with a TB-sensitive approach, alone they would be insufficient to prevent DS TB-related catastrophic costs in any of the 6 countries, and when increased enough to prevent DS TB catastrophic costs would require a budget between $298 million (95%CI: $219-$378 million) and $165 billion (95%CI: $134-$196 billion) per country. DR TB-related costs were catastrophic before and after TB-specific or TB-sensitive cash transfers in 1/1 countries. Sensitivity analyses showed our findings to be robust to imputation of missing TB-related cost components, and use of 10% or 30% instead of 20% as the threshold for measuring catastrophic costs. Key limitations were using national average data, and not considering other health and social benefits of cash transfers.  Conclusions. A TB-sensitive cash transfer approach to increase all poor households’ income may have broad benefits by reducing poverty, but is unlikely to be as effective or affordable for preventing TB catastrophic costs as a TB-specific cash transfer approach to defray TB-related costs only in poor households with a confirmed TB diagnosis. Preventing DR TB-related catastrophic costs will require considerable additional investment whether a TB-sensitive or a TB-specific cash transfer approach is used" |
| **Introduction** |  |  |  |
| Background and objectives | 3 | Provide an explicit statement of the broader context for the study. Present the study question and its relevance for health policy or practice decisions. | Introduction, paragraph 4: "Depending on whether cash transfers are provided with a TB-specific or a TB-sensitive approach, their impact might vary. We aimed to investigate how this might relate to the potential of cash transfers provided with either a TB-specific approach or a TB-sensitive approach to prevent catastrophic costs." |
| **Methods** |  |  |  |
| Target population and subgroups | 4 | Describe characteristics of the base case population and subgroups analysed, including why they were chosen. | Methods, paragraph 4: "In this study, the target population for cash transfers provided with a TB-specific approach was households in countries’ poorest population quintile with a confirmed TB diagnosis. Guidance is not currently available for which TB-affected households should be targeted with a TB-specific approach. We chose to focus on TB-affected households in countries’ poorest population quintile because they are typically at greater risk of incurring catastrophic costs (14). Whilst it might have been preferable to focus on all TB-affected households that incur catastrophic costs, at the time of analysis no estimates of the size of this population were available in any countries included in this study. The target population for cash transfers provided with a TB-sensitive approach was households in poverty already targeted by countries’ established governmental poverty-reduction cash transfer programme." |
| Setting and location | 5 | State relevant aspects of the system(s) in which the decision(s) need(s) to be made. | Methods, paragraph 1: "The setting was low- and middle-income countries, where over 95% of TB cases live, and where formal institutions to protect households from the social and economic impacts of illness are weakest." |
| Study perspective | 6 | Describe the perspective of the study and relate this to the costs being evaluated. | Methods, paragraph 1: "Using only TB-related costs incurred by patients, study outcomes were assessed from the patient perspective." |
| Comparators | 7 | Describe the interventions or strategies being compared and state why they were chosen. | Methods, paragraph 1: "The intervention being investigated was cash transfers paid to poor households in low- and middle-income countries, and the alternative approaches being compared were cash transfers provided with either a TB-specific or a TB-sensitive approach. These approaches were compared because of current uncertainty about the impact and country-level cost of each approach." |
| Time horizon | 8 | State the time horizon(s) over which costs and consequences are being evaluated and say why appropriate. | Methods, paragraph 2: "Catastrophic costs were estimated over a time horizon from the month prior to TB symptom onset to TB treatment completion. Countries’ country-level cash transfer budget were estimated over a time horizon of one year." |
| Discount rate | 9 | Report the choice of discount rate(s) used for costs and outcomes and say why appropriate. | This study did not use a discount rate for costs. |
| Choice of health outcomes | 10 | Describe what outcomes were used as the measure(s) of benefit in the evaluation and their relevance for the type of analysis performed. | Methods, paragraph 2: "Primary study outcomes were an indicator for catastrophic costs after TB-specific versus TB-sensitive cash transfers, and countries’ country-level cash transfer budget needed to prevent catastrophic costs for each of these approaches." |
| Measurement of effectiveness | 11a | Single study-based estimates: Describe fully the design features of the single effectiveness study and why the single features of the single effectiveness study and why the single study was a sufficient source of clinical effectiveness data. | This study used single study-based estimates of average TB-related costs, cash transfers and household income as input parameters for the seven countries included in the analysis. Methods, paragraph 5 describes how included TB-related cost data were identified by systematic review. Results, paragraph 2 and 3 provides a summary of TB-related cost data sources used in the study  Methods, paragraph 6 describes how included cash transfer data were identified by rigorous review of data from the World Bank Atlas of Social Protection Indicators of Resilience and Equity. S1 Table provides a summary of cash transfer data sources used in the study.  Methods, paragraph 7 describes how included household income data were identified by rigorous search of countries’ national statistical websites and the international household survey network website. S1 Table provides a summary of household income data sources used in the study. |
|  | 11b | Synthesis-based estimates: Describe fully the methods used for identification of included studies and synthesis of clinical effectiveness data. | This study did not use synthesis based estimates for its input parameters. |
| Measurement and valuation of preference based outcomes | 12 | If applicable, describe the population and methods used to elicit preferences for outcomes. | This study did not attempt to elicit preferences for outcomes. |
| Estimating resources and costs | 13a | Single study-based economic evaluation: Describe approaches used to estimate resource use associated with the alternative interventions. Describe primary or secondary research methods for valuing each resource item in terms of its unit cost. Describe any adjustments made to approximate to opportunity costs. | This study was not a single study-based economic evaluation. |
|  | 13b | Model-based economic evaluation: Describe approaches and data sources used to estimate resource use associated with model health states. Describe primary or secondary research methods for valuing each resource item in terms of its unit cost. Describe any adjustments made to approximate to opportunity costs. | Resource use, value and opportunity costs associated with model health states were not estimated in this study. |
| Currency, price date, and conversion | 14 | Report the dates of the estimated resource quantities and unit costs. Describe methods for adjusting estimated unit costs to the year of reported costs if necessary. Describe methods for converting costs into a common currency base and the exchange rate. | Methods, paragraph 10 describes methods for adjusting included monetary data to a common year and converting it into a common currency: "To allow comparison of monetary data extracted in different currencies and measured in different years all extracted monetary values were inflated and converted to 2013 international dollars ($) using the purchasing power parity conversion factor that accounts for differences in the cost of living across countries." |
| Choice of model | 15 | Describe and give reasons for the specific type of decision analytical model used. Providing a figure to show model structure is strongly recommended. | The analytical model used in this study is represented by the quantitative relationship between TB-related costs, household income and cash transfers depending on whether cash transfers are provided with a TB-specific or a TB-sensitive approach. Methods, paragraphs 13-17, give reasons for the specific for the specific type of decision analytical model used. Box 2 provides a summary of how the relationships between TB-related costs, household income and cash transfers is modelled depending on whether cash transfers are provided with a TB-specific or a TB-sensitive approach |
| Assumptions | 16 | Describe all structural or other assumptions underpinning the decision-analytical model. | Assumptions were made about the size of countries’ TB-specific target population. Methods, paragraph 8 describes this: "Because estimates of the percentage of TB-affected households represented in the poorest population quintile were not available in any countries included in the study, we used the unweighted mean multiplier for TB prevalence in the poorest population quintile observed in India and South Africa to estimate the size of countries’ TB-specific target population. Therefore, countries TB-specific target population was extracted as 40% of countries’ estimated 2013 DS TB burden or 2015 DR TB burden. For country estimates of DR TB burden, we used 2015 estimates because 2013 estimates weren’t available. We assumed that each estimated case of active TB disease in the World Health Organisation’s TB data represented one household with a confirmed TB diagnosis."  Assumptions were also made about the value of missing cost components of countries TB-related costs. Methods, paragraph 11 describes this: "In countries that had missing values for direct or indirect costs, pre- or during-treatment we estimated their value. To do this, we assumed that average TB-related costs followed a make-up of cost components equivalent to the one synthesised by Tanimura *et al.* in their systematic review of TB-related costs in low- and middle-income countries, which is that direct and indirect costs are equivalent to 40% and 60% of total costs respectively, and pre- and during-treatment costs are each equivalent to 50% of total costs respectively"  An assumption was made about countries’ average household income value. Methods, paragraph 7 describes this: "For countries with an eligible TB-related cost survey and existing poverty-reduction cash transfer programme, we used countries’ mean household income or expenditure in the poorest population quintile to approximate household income of both TB-specific and TB-sensitive target populations."  Assumptions were made about the standard deviation of included estimates of countries average TB-related costs, cash transfers, and household income. Methods, paragraph 12 describes this: "Random samples were generated for TB-related costs using a standard deviation with a ratio of 1.1 to mean estimates, which was the ratio estimated by Tanimura *et al.* for average total costs across all low- and middle-income countries, and a sample size equivalent to countries’ cost surveys [7]. For annual household income, we used a standard deviation with a ratio of 0.8 to mean estimates, which was the average observed across two studies investigating the household-level income effect of poverty-reduction cash transfer programmes in Brazil and Colombia [37,44], and a sample size equivalent to countries’ household income surveys [55–61]. For cash transfers, we used a standard deviation with a ratio to mean estimates equivalent to a quarter of maximum minus minimum cash transfers, and a sample size equivalent to the one reported in studies from which we extracted mean cash transfers. In Ecuador and Ghana, we did not simulate sampling distributions for cash transfers because respectively, all beneficiary households receive the same flat cash transfer, and the mean cash transfer we extracted was estimated from all beneficiary households. " |
| Analytical methods | 17 | Describe all analytical methods supporting the evaluation. This could include methods for dealing with skewed, missing, or censored data; extrapolation methods; methods for pooling data; approaches to validate or make adjustments (such as half cycle corrections) to a model; and methods for handling population heterogeneity and uncertainty. | In this study, missing data was interpolated. Methods, paragraph 11 describes how this was done: "In countries that had missing values for direct or indirect costs, pre- or during-treatment we estimated their value. To do this, we assumed that TB-related costs followed a make-up of cost components equivalent to the one synthesised by Tanimura et al. in their systematic review of TB-related costs in low- and middle-income countries, which is that direct and indirect costs are equivalent to 40% and 60% of total costs respectively, and pre- and during-treatment costs are each equivalent to 50% of total costs respectively."  In this study a multiway analysis was used to account for uncertainty in input parameters. Methods, paragraph 12 describes how this was done: "To account for uncertainty in the value of extracted TB-related costs, annual household income and cash transfers, we conducted a multiway analysis that allowed all three of these inputs to vary simultaneously according to their sampling distributions. Sampling distributions were simulated from 10,000 computationally generated random samples, and were all assumed normal according to the central limit theorem."  In this study, sensitivity analyses were run to test the sensitivity of results to imputation of missing data, and the use of a 20% threshold for defining catastrophic costs. Methods, paragraph 18 describes how this was done: "We tested the sensitivity of our results in Brazil, Colombia, Tanzania and Mexico to imputation of missing TB-related cost components by repeating our analysis omitting rather than imputing the value of missing TB-related cost components. We separately tested the sensitivity of our results across all countries included in the study to the use of 20% as the threshold for measuring countries’ TB-related cost burden as catastrophic. We did this by repeating our analyses instead using a 10% and 30% threshold." |
| **Results** |  |  |  |
| Study parameters | 18 | Report the values, ranges, references, and, if used, probability distributions for all parameters. Report reasons or sources for distributions used to represent uncertainty where appropriate. Providing a table to show the input values is strongly recommended. | Table 1, Table 2 and S1 Table report values, 95% confidence intervals or ranges, and probability distributions with reasoning for all TB-related cost, cash transfers and household income data used in the study. |
| Incremental costs and outcomes | 19 | For each intervention, report mean values for the main categories of estimated costs and outcomes of interest, as well as mean differences between the comparator groups. If applicable, report incremental cost-effectiveness ratios. | Figure 2 and Table S3 report included countries TB-related cost burden before cash transfers, and after TB-specific or TB-sensitive cash transfers. Table 3 reports included countries’ household-level additional and total cash transfer needed to prevent catastrophic costs. Figure 3 and Table S4 report included countries’ total TB-specific or TB-sensitive country-level cash transfer budget needed to prevent catastrophic costs. |
| Characterising uncertainty | 20a | Single study-based economic evaluation: Describe the effects of sampling uncertainty for the estimated incremental cost and incremental effectiveness parameters, together with the impact of methodological assumptions (such as discount rate, study perspective). | This study was not a single study-based economic evaluation. |
|  | 20b | Model-based economic evaluation: Describe the effects on the results of uncertainty for all input parameters, and uncertainty related to the structure of the model and assumptions. | Results, paragraph 11 describes the effects of imputation of missing cost components on the results: "Before cash transfers, the TB-related cost burden remained catastrophic in the same countries as when missing TB-related cost components were imputed, and the only difference after cash transfers was that TB-specific cash transfers prevented catastrophic costs in Colombia, S4 Table. Across countries, TB-specific cash transfers remained more affordable at preventing catastrophic costs compared to TB sensitive cash transfers both at the household and country level, S5 Table."  Results, paragraph 12 describes the effects of using a 20% threshold TB-related cost burden for measuring catastrophic costs instead of a 10% threshold: "Before cash transfers, in addition to Colombia, Ecuador, Ghana, Mexico, Tanzania and Yemen, the DS TB-related cost burden was also catastrophic in Brazil. In Ecuador, the DR TB-related cost burden before cash transfers remained catastrophic. Across countries, TB-specific cash transfers remained more affordable than TB-sensitive cash transfers at preventing DS and DR TB catastrophic costs both at the household and country level, S6 Table. "  Results, paragraph 13 describes the effects of using a 20% threshold TB-related cost burden for measuring catastrophic costs instead of a 30% threshold: "Before cash transfers, the DS TB-related cost burden remained catastrophic in Colombia, Ghana, Mexico, Tanzania and Yemen, but ceased to be catastrophic in Ecuador. In Ecuador, the DR TB-related cost burden before cash transfers remained catastrophic. Across countries, TB-specific cash transfers remained more affordable than TB-sensitive cash transfers at preventing DS and DR TB catastrophic costs both at the household and country level, Table S8: Summary of countries’ household-level additional and total cash transfer, and country-level cash transfer budget needed to prevent catastrophic costs using a 30% threshold TB-related cost burden for measuring catastrophic costs." |
| Characterising heterogeneity | 21 | If applicable, report differences in costs, outcomes, or cost-effectiveness that can be explained by variations between subgroups of patients with different baseline characteristics or other observed variability in effects that are not reducible by more information. | This study considered differences in costs and outcomes between patients with drug-susceptible and drug-resistant TB. Findings and their implications are consistently reported separately for these two subgroups. |
| **Discussion** |  |  |  |
| Study findings, limitations, generalisability, and current knowledge | 22 | Summarise key study findings and describe how they support the conclusions reached. Discuss limitations and the generalisability of the findings and how the findings fit with current knowledge. | Discussion, paragraphs 1 and 2 summarise key study findings. Discussion, paragraph 3 describes how the study’s findings support the conclusions reached. Discussion, paragraph 5 discusses the study’s limitations. Discussion, paragraph 6 discusses how the study’s findings fit with current knowledge. |
| **Other** |  |  |  |
| Source of funding | 23 | Describe how the study was funded and the role of the funder in the identification, design, conduct, and reporting of the analysis. Describe other non-monetary sources of support. | The financial disclosure field describes how the study was funded: "This research and members of the research team were funded by the Medical Research Council (award MR/K006584/1); the charity Innovation For Health And Development (IFHAD); the Joint Global Health Trials consortium (MRC, DFID, & Wellcome Trust award MR/K007467/1); the Bill and Melinda Gates Foundation (award OPP1118545); TB REACH and the Wellcome Trust (award 104473/Z/14/Z). The funders had no role in study design, data collection and analysis, decision to publish, or preparation of the manuscript." |
| Conflicts of interest | 24 | Describe any potential for conflict of interest of study contributors in accordance with journal policy. In the absence of a journal policy, we recommend authors comply with International Committee of Medical Journal Editors recommendations. | Conflicting interests are described in the competing interests field with the disclaimer: "All authors have declared that they have no competing interests. CAE notes that he is an academic editor for PLOS Medicine." |
