# Supplementary material for: Comparison of two cash transfer strategies to prevent catastrophic costs for poor tuberculosis-affected households in low- and middle-income countries: An economic modelling study
Source: PLoS Med. 2017 Nov 7;14(11):e1002418. doi: 10.1371/journal.pmed.1002418 (PMC5675360; doi:10.1371/journal.pmed.1002418)
Supplement: S1 Text — (DOCX) [file pmed.1002418.s002.docx]

**Study title:** Mitigating Catastrophic Costs of TB Patients; The Role of Cash Transfers for TB Control

**Study aim:** Assess the potential of cash transfer programmes to mitigate the financial cost of seeking care for TB patients in low and middle income countries

**Study objectives:**

1. Review and quantify the mitigation effect of national cash transfer programs on costs incurred by Tuberculosis (TB) and Multi Drug Resistant (MDR)-TB patients
2. Qualitatively analyse the potential of national cash transfer programs in Brazil and Peru to mitigate costs to TB patients, and explore issues relating to their implementation
3. Qualitatively analyse the potential of national cash transfer programs in Brazil and Peru to become more inclusive of TB patients using relevant targeting criteria
4. Generate and identify evidence gaps that might be used to inform the design of future prospective studies to better understand the role of cash transfers in mitigating the catastrophic costs incurred by impoverished TB patients

**Study hypothesis:** TB disproportionately affects poor and vulnerable individuals who are unable to afford the financial burden of seeking care and completing treatment. By reducing patient’s ability to work TB disease also reduces sources of available household income. Because of these factors many TB patients are either forced to sell assets and take out loans to pay for treatment, or seek informal sources income to pay for treatment. Ultimately these financial consequences have an important impact on patients’ ability to adhere to treatment. Cash transfers might have the potential to mitigate the direct and indirect costs to patients with TB or MDR-TB and break the poverty trap associated with TB disease. Our hypothesis is that this mitigation effect may vary depending on the cash transfer implementation strategy and on whether the cash transfer is TB sensitive, TB-inclusive or TB-specific, Box 1.

**Box 1: Definitions of three potential cash transfer schemes for TB control**

**TB-specific initiatives:** Cash transfer interventions explicitly targeting TB-affected individuals and/or households with the intent of addressing a specific TB care and prevention issue.

**TB-inclusive initiatives:** Cash transfer schemes that are not limited to TB-related issues but amongst their objectives include explicit TB care and support.

**TB-sensitive initiatives:** Cash transfers interventions not specific to TB patients but that could have an impact for TB patients or for TB prevention because they target groups and/or people at high risk of TB and vulnerable to deeper impoverishment due to its consequences.

**Study background:** TB disproportionately affects the most vulnerable segments of society and often prevents employment, thereby exacerbating the poverty level of TB-affected households (83). Despite most National TB control programs (NTPs) offering “free” and “equitable” health care, the financial burden of hidden direct and indirect costs to poor TB affected households often requires that families dedicate catastrophic proportions of their available income on seeking care. A threshold of 10%–25% of patients’ household annual income is deemed to be excessive and a “catastrophic” burden to TB patients [1,2]. According to a recent review of patient costs in low- and middle-income countries, poor TB patients experience costs equivalent to an average of 39% of reported household income whilst seeking care [3].

The first milestone of the End TB Strategy is to protect all TB-affected families from facing catastrophic costs by 2025. Moreover the second pillar of the strategy —“bold policies and supportive systems”— acknowledges social protection as an important tool to achieve this target [4]. Social protection has been defined as a range of policies that enable people to cope with and recover from risks and adversities, with the objective of achieving poverty reduction and sustainable and inclusive economic growth [5]. The most popular forms of social protection are cash transfer schemes that provide cash to poor population groups to reduce vulnerability and poverty. Cash transfers can be given unconditionally or conditionally requiring recipients to take specific behavioural, education or health actions prior to cash being transferred [5].

Due to their proven impact on human and financial capital, cash transfers are now an integral part of the response to children malnutrition, maternal health and HIV/AIDS [6]. As for HIV/AIDS, cash transfer interventions have the potential to strengthen TB control by: 1) enhancing TB prevention; 2) supporting the access to TB diagnosis and care; 3) protecting TB patients from financial vulnerability simultaneously promoting longer term recovery from poverty. However, there is still limited evidence to support the potential of these interventions to contribute to reducing the financial vulnerability of TB patients [7].

This study will attempt to partially fill this knowledge gap by describing the potential mitigation effect of existing cash transfer schemes if TB-affected households were enrolled in these schemes. To achieve this cash benefits provided by a list of selected cash transfer schemes in various countries will be systematically reviewed and compared with the known costs faced by TB patients in the same countries. This quantitative component will be complemented with an in-depth analysis of the opportunities for a cross-sectoral partnership between NTPs in Peru and Brazil, and the two ongoing national cash transfer programs in these countries: Juntos program in Peru, and Bolsa Familia program in Brazil. This component will highlight important operational issues for TB sensitive programs, and will explore whether other more targeted cash transfer programs might be more suitable for TB control in the future.

**Study methods:** This study will focus on low- and middle- income countries for which a) TB and or MDR-TB costs are available, and b) there is a recognised ongoing cash transfer program. Based on these criteria the study will systematically review the literature to assess the potential of cash transfer schemes to mitigate catastrophic costs associated with TB care seeking. This study will use the definition of “total costs corresponding to ≥20% of annual household income”, proposed as possible threshold for catastrophic costs [2]. This quantitative review will be complemented by a qualitative situational analysis using key informant interviews to provide a more in-depth analysis of the potential mitigation effect of TB-specific and TB-sensitive cash transfer schemes.

*Data source and search strategy:* In order to gather information on costs incurred by TB patients this study will include studies identified by the recent systematic review of TB patient costs [3]. The original search will be updated using the same strategy to include studies written in English conducted in low- and middle- income countries and published from March 31, 2013, to March 31, 2015. Studies will be excluded from the updated search according to the original exclusion criteria [3].

For the updated search the same electronic databases used by Tanimura et al. will be searched including Pubmed; Global Information Full text; Index Medicus for Africa; South East Asia; Eastern Mediterranean region and Western Pacific Region; and Literature Latinoamericana y del Caribe en Ciencias de la Salud. The search terms will be “tuberculosis” (tuberculosis, TB, or tuberculosis as a MeSh Term in PubMed), and “cost” (cost(s), expense(s), economic, expenditure(s), payment(s), out-of-pocket, financial, impoverishment, or catastrophic).

Benefits provided by cash transfer programmes will be sourced using the most comprehensive publicly available publications and programme-related documents obtained from international and governmental agency websites, including sources such as the World Bank, UNDP, and National Ministries.

*Data extraction:* Background study information will be extracted to generate a synopsis table of studies meeting the review’s inclusion criteria. The following background information will be extracted: authors, country, study type, study objective, year of the publication, setting characteristics, type of TB, name and category of cash transfer scheme implemented in the country from which TB costs data are provided and the average size of the cash benefit provided. Data on the mean costs incurred by TB patients will also be extracted and will include: direct medical costs (e.g., consultations, tests and medicines), direct non-medical costs (e.g., transport and food during healthcare visits), and indirect costs (e.g., lost income). Wherever possible costs will be stratified by DS TB or DR TB.

*Data analysis:* Data collected from the systematic review of countries meeting the review’s eligibility criteria will be used to estimate the potential of cash transfers to mitigate total costs to below the catastrophic threshold of 20% of TB patient’s annual household income. This analysis will consist of two stages.

First, the total value of direct and indirect costs to patients will be calculated as a percentage of annual household income before receipt of cash benefits. This part of the analysis will reveal countries where TB patients incur average costs above the catastrophic threshold of 20% of annual household income.

Secondly, the total value of direct and indirect costs to patients as a percentage of annual household income after hypothetical receipt of cash benefits will be used to assess the potential of existing cash transfers to mitigate average costs to below 20% of TB patient’s annual household income.

*Key informant interviews:* The primary objective of this situational analysis will be to ask experienced professionals to better describe the actual situation of TB control, and costs incurred by patients. The analysis will also speculate on the potential mitigation effect of cash transfer schemes under two different implementation scenarios. The countries chosen for this analysis were Peru and Brazil, with Peru representing the most comprehensive on-going TB-specific cash transfer scheme, and Brazil an on-going national social protection programme with the greatest potential to be TB-sensitive [8].

CRESIPT is a community randomised study in Peru that will aim to provide rigorous evidence of the impact of conditional cash transfers to mitigate adverse social and financial consequences of TB patients. CRESIPT has been preceded by a recently ended pilot phase to implement and refine the complex socioeconomic intervention in 32 communities; assess its impact on TB chemoprophylaxis completion, and assess its acceptance through a process evaluation. The results of this pilot study have informed the design of the subsequent 6-year study that starts in September 2015.

Bolsa Familia is a social protection scheme of the Brazilian government that provides conditional cash transfers to poor Brazilian families. Brazil is considered as a form of best practice, sustainable, scalable social protection intervention for TB control [8]. However, much work needs to be done to improve the proportion of TB patients that actually receive benefits from Bolsa Familia as a 2010 analysis in Brazil showed that only 14% of TB patients who live below the poverty line receive benefits from Bolsa Familia.

For Peru, principal and co-investigators of CRESIPT will be interviewed, as well as informants from the Peruvian NTP. For Brazil informants will include the Director of the NTB programme, BFP representatives from the Ministry of Development, and national scientists working on the impact of BFP on TB in Brazil.

In order to conduct this analysis data will be collected from key informants using a formal semi-structured questionnaire. Key informants will be recruited by email invitation. The invitation will include information about the purpose of the study, the scope of the study, the types of questions which are likely to be asked, as well as the use to which the results will be put.

Wherever possible interviews will be conducted in person. No questions relating to individual level data will be asked. Written consent will be obtained from respondents before formally starting the interview to ensure that they agree to disclose the information, and agree to have it published later in possible scientific publications. All interviews will be recorded for transcription purposes as well as support the depth of data collection. Interviews will be complemented with relevant “grey literature”. The transcripts will be coded manually and identified themes will be fed back to interviewees for validation.

**References**

1. Laokri S, Weil O, Drabo KM, Dembelé SM, Kafando B, Dujardin B. Removal of user fees no guarantee of universal health coverage: observations from Burkina Faso. Bull World Health Organ. 2013;91: 277–282. doi:10.2471/BLT.12.110015

2. Wingfield T, Boccia D, Tovar M, Gavino A, Zevallos K, Montoya R, et al. Defining Catastrophic Costs and Comparing Their Importance for Adverse Tuberculosis Outcome with Multi-Drug Resistance: A Prospective Cohort Study, Peru. PLoS Med. 2014;11: e1001675. doi:10.1371/journal.pmed.1001675

3. Tanimura T, Jaramillo E, Weil D, Raviglione M, Lönnroth K. Financial burden for tuberculosis patients in low- and middle-income countries: a systematic review. Eur Respir J. 2014;43: 1763–1775. doi:10.1183/09031936.00193413

4. World Health Organization. End TB Strategy [Internet]. Geneva: World Health Organization; 2015. Available: http://www.who.int/entity/tb/post2015_TBstrategy.pdf

5. Adato M, Bassett L. Social protection to support vulnerable children and families: the potential of cash transfers to protect education, health and nutrition. AIDS Care. 2009;21: 60–75. doi:10.1080/09540120903112351

6. Lutz B, Small R. Discussion Paper: Cash Transfers and HIV Prevention [Internet]. New York: UNDP; 2014 Oct. Available: http://www.undp.org/content/dam/undp/library/HIV-AIDS/HIV%20MDGs%20and%20Development%20Planning/UNDP%20Cash%20Transfers%20and%20HIV%20Prevention%20Web%20Final.pdf?download

7. Rocha C, Montoya R, Zevallos K, Curatola A, Ynga W, Franco J, et al. The Innovative Socio-economic Interventions Against Tuberculosis (ISIAT) project: an operational assessment. Int J Tuberc Lung Dis Off J Int Union Tuberc Lung Dis. 2011;15: S50–S57. doi:10.5588/ijtld.10.0447

8. Chatham House. Social Protection Interventions for Tuberculosis Control: The Impact, the Challenges and the Way Forward [Internet]. Chatham House; 2012. Available: https://www.chathamhouse.org/sites/files/chathamhouse/public/Research/Global%20Health/170212summary.pdf
