# Supplementary material for: Comparison of two cash transfer strategies to prevent catastrophic costs for poor tuberculosis-affected households in low- and middle-income countries: An economic modelling study
Source: PLoS Med. 2017 Nov 7;14(11):e1002418. doi: 10.1371/journal.pmed.1002418 (PMC5675360; doi:10.1371/journal.pmed.1002418)
Supplement: S1 Table — PPP, purchasing power parity. (DOCX) [file pmed.1002418.s003.docx]

| **Country** |  | **Cash transfer data** | | | |  | | **Household income data** | | | | |
| --- | --- | --- | --- | --- | --- | --- | --- | --- | --- | --- | --- | --- |
|  |  | **Poverty-reduction cash transfer programme** | **Year of reported data** | **Sample size, in households** |  | | **Household income or expenditure survey** | | **Year of reported data** | **Sample size, in households** | **Household income**† **2013 PPP$** |  |
| Brazil |  | Programa Bolsa Familia [1,2] | 2008 and 2013 | 9,149 |  | | Pesquisa de Orçamentos Familiares [3] | | 2008 | 55,970 | 5,331 |  |
| Ecuador |  | Bono de Desarollo  Humano [4] | 2013 | N.A. |  | | Encuesta Nacional de Ingresos y Gastos de los Hogares Urbanos y Rurales [5] | | 2011 | 40,932 | 8,692 |  |
| Yemen |  | Social Welfare Fund [6] | 2013 | 3,886 |  | | Household Budget Survey [7] | | 2005 | 13,136 | 2,143 |  |
| Tanzania |  | Productive Social Safety Net [8–10] | 2013, 2014 and 2015 | 892 |  | | Household Budget Survey‡ [11] | | 2007 | 10,466 | 2,812 |  |
| Ghana |  | Livelihood Empowerment Against Poverty [12] | 2014 | 72,400 |  | | Ghana Living Standards Survey-Round Five [13] | | 2006 | 8,687 | 1,785 |  |
| Colombia |  | Mas Familias en Accion [14,15] | 2005 and 2013 | 2,804 |  | | Encuesta Nacional de Ingresos y Gastos de los Hogares [16] | | 2006 | 42,733 | 2,214 |  |
| Mexico |  | Oportunidades* [17,18] | 2004 and 2013 | 5,055 |  | | Encuesta Nacional de Ingresos y Gastos de los Hogares [19] | | 2010 | 30,169 | 4,755 |  |

*Formerly PROGRESA. †Refers to mean annual household income in countries’ poorest population quintile. ‡Survey only reported mean household expenditure in country’s poorest population quintile

**References**

1. Social Development Division, Economic Commission for Latin America and the Caribbean of the United Nations (ECLAC). Bolsa Familia (2003-) - Conditional Cash Transfer Programmes - Non-contributory social protection programmes in Latin America and the Caribbean database [Internet]. 2017 [cited 22 Jul 2017]. Available: http://dds.cepal.org/bdptc/en/program/?id=6#

2. Cruz M, Ziegelhofer Z. Beyond the income effect: impacts of conditional cash transfer programs on private investments in human capital [Internet]. Washington, DC: World Bank Group; 2014 May p. 111. Report No.: WPS6867. Available: http://documents.worldbank.org/curated/en/2014/05/19520425/beyond-income-effect-impacts-conditional-cash-transfer-programs-private-investments-human-capital

3. Instituto Brasileiro de Geografia e Estatística (IBGE). Pesquisa de Orçamentos Familiares 2008-2009: Despesas, rendimentos e condições de vida [Internet]. [cited 21 Jul 2016]. Available: ftp://ftp.ibge.gov.br/Orcamentos_Familiares/Pesquisa_de_Orcamentos_Familiares_2008_2009/Despesas_rendimentos_e_condicoes_de_vida/tab_rendimentos.zip

4. Social Development Division, Economic Commission for Latin America and the Caribbean of the United Nations (ECLAC). Bono de Desarrollo Humano (Human Development Grant) (2003-) - Conditional Cash Transfer Programmes - Non-contributory social protection programmes in Latin America and the Caribbean database [Internet]. 2017 [cited 22 Jul 2017]. Available: http://dds.cepal.org/bdptc/en/program/?id=15

5. Instituto Nacional de Estadística y Censos. Encuesta Nacional de Ingresos y Gastos de los Hogares Urbanos y Rurales [Internet]. [cited 21 Jul 2017]. Available: http://www.ecuadorencifras.gob.ec//documentos/web-inec/Estadisticas_Sociales/Encuesta_Nac_Ingresos_Gastos_Hogares_Urb_Rur_ENIGHU/ENIGHU-2011-2012/TABULADOS%20ENIGHUR%202011-2012.xlsx

6. Yemen National Social Protection Monitoring Survey (NSPMS): 2012-2013 Final Report [Internet]. Brasilia: IPC-IG, UNICEF Yemen; 2014 Dec. Available: http://www.ipc-undp.org/pub/eng/Yemen_National_Social_Protection_Monitoring_Survey_2012_2013.pdf

7. Yemen, Rep. Central Statistical Organization. Household Budget Survey 2005-2006 [Internet]. [cited 15 Nov 2016]. Available: http://catalog.ihsn.org/index.php/catalog/230

8. Tanzania Government Project Preparation Team. Productuve Social Safety Net (PSSN) Operational Manual [Internet]. Dar es Salaam; 2013 Jan. Available: http://www.tasaf.go.tz/index.php/reports/tasaf-documemt-archive/publications/158-tasaf-iii-pssn-operational-manual/file

9. Tanzania Social Action Fund. Tanzania Social Action Fund Condtional Cash Transfer [Internet]. 2017 [cited 30 Aug 2015]. Available: http://tasaf.org/index.php/tasaf-components/supported-interventions/conditional-cash-transfer

10. Evans DK, Hausladen S, Kosec K, Reese N. Community based conditional cash transfers in Tanzania: results from a randomized trial [Internet]. Washington, DC: The World Bank; 2014 Mar. Available: https://doi.org/10.1596/978-1-4648-0141-9

11. Tanzania National Bureau of Statistics. National Household Budget Survey 2007 [Internet]. [cited 21 Jul 2017]. Available: http://www.ilo.org/surveydata/index.php/catalog/508/download/5091

12. van de Meerendonk A, Behrendt C, Kauffmann VW. Rationalizing social protection expenditure in Ghana [Internet]. Geneva: International Labour Organization; 2015. Available: http://www.ilo.org/wcmsp5/groups/public/---ed_protect/---soc_sec/documents/publication/wcms_431794.pdf

13. Ghana Statistical Service. Ghana Living Standard Survey 5: 2005 [Internet]. Accra: Ghana Statistical Service; 2008 Sep. Available: http://www.statsghana.gov.gh/docfiles/glss5_report.pdf

14. Ospina M. The Indirect Effects of Conditional Cash Transfer Programs: An Empirical Analysis of Familias En Accion [Internet]. Dissertation, Georgia State University. 2010. Available: http://scholarworks.gsu.edu/cgi/viewcontent.cgi?article=1059&context=econ_diss

15. Social Development Division, Economic Commission for Latin America and the Caribbean of the United Nations (ECLAC). Más Familias en Acción (More Families in Action) (2001-) - Conditional Cash Transfer Programmes - Non-contributory social protection programmes in Latin America and the Caribbean database [Internet]. 2017 [cited 22 Jul 2017]. Available: http://dds.cepal.org/bdptc/en/program/?id=12

16. Departamento Administrativo Nacional De Estadística (DANE). Encuesta Nacional de Ingresos y Gastos de los Hogares [Internet]. [cited 15 Nov 2016]. Available: https://www.dane.gov.co/files/investigaciones/condiciones_vida/ingresos_gastos/Cuadros_Total_Nacional_ENIG0607.xls

17. Social Development Division, Economic Commission for Latin America and the Caribbean of the United Nations (ECLAC). Oportunidades (Human Development Programme, formerly “Progresa“) (1997-2014) - Conditional Cash Transfer Programmes - Non-contributory social protection programmes in Latin America and the Caribbean database [Internet]. 2017 [cited 22 Jul 2017]. Available: http://dds.cepal.org/bdptc/en/program/?id=22

18. Angelucci MA, Attanasio O, Di Maro V. The Impact of Oportunidades on Consumption, Savings and Transfers. Fisc Stud. 2012;33: 305–334. doi:10.1111/j.1475-5890.2012.00163.x

19. Instituto Nacional de Estadística y Geografía (INEGI). Encuesta Nacional de Ingresos y Gastos de los Hogares (ENIGH) 2010 Nueva construcción [Internet]. [cited 15 Nov 2016]. Available: http://www.beta.inegi.org.mx/contenidos/proyectos/enchogares/regulares/enigh/nc/2010/tabulados/tabulados_2010_ncv.xls
