# Supplementary material for: Comparison of two cash transfer strategies to prevent catastrophic costs for poor tuberculosis-affected households in low- and middle-income countries: An economic modelling study
Source: PLoS Med. 2017 Nov 7;14(11):e1002418. doi: 10.1371/journal.pmed.1002418 (PMC5675360; doi:10.1371/journal.pmed.1002418)
Supplement: S2 Table — The “Before cash transfers” column represents countries’ mean TB-related cost burden without cash transfer data. The “After TB-specific cash transfers” column represents countries’ mean TB-related cost burden after cash transfers have been subtracted from TB-related costs. The “After TB-sensitive cash transfers” column represents countries’ mean TB-related cost burden after cash transfers have been added to countries’ pre-illness household income. CI, confidence interval; DR, drug-resistant; DS, drug-susceptible; TB, tuberculosis. (DOCX) [file pmed.1002418.s004.docx]

|  |  | **TB-related cost burden (% of annual household income) *** | | |
| --- | --- | --- | --- | --- |
| **Country** |  | **Before cash transfers (95% CIs)** †‡ | **After TB-specific cash transfers (95% CIs)** †‡ | **After TB-sensitive cash transfers (95% CIs)** †‡ |
| **DS TB** |  |  |  |  |
| Brazil |  | 15 (12-18) | 0.0 (0.0-2.0) | 13 (10-15) |
| Ecuador |  | 27 (21-32) | 14 (8.6-20) | 24 (19-29) |
| Yemen |  | 41 (36-46) | 0.0 (0.0-3.3) | 28 (25-32) |
| Tanzania |  | 59 (46-73) | 51 (38-63) | 55 (43-67) |
| Ghana |  | 68 (55-80) | 42 (30-55) | 54 (44-64) |
| Colombia |  | 64 (53-75) | 26 (15-38) | 46 (38-55) |
| Mexico |  | 125 (105-145) | 106 (86-126) | 105 (88-121) |
| **DR TB** |  |  |  |  |
| Ecuador |  | 192 (86-299) | 179 (74-286) | 170 (77-265) |

*Household income refers to average household income in the poorest population quintile. †For interpretability, negative estimates and confidence intervals were reported as 0. ‡To estimate 95% confidence intervals, all mean TB-related costs were assumed to have a standard deviation with a ratio of 1.1 to their value [1], all mean household incomes were assumed to have a standard deviation with a ratio of 0.8 to their value [2,3](36,39), and all mean cash transfers were assumed to have a standard deviation equal to a quarter of maximum minus minimum cash transfers. Probability distributions for all three input parameters were assumed to be normal. This was justified because our analysis was at the national level and we used mean values.

**References**

1. Tanimura T, Jaramillo E, Weil D, Raviglione M, Lönnroth K. Financial burden for tuberculosis patients in low- and middle-income countries: a systematic review. Eur Respir J. 2014;43: 1763–1775. doi:10.1183/09031936.00193413

2. Cruz M, Ziegelhofer Z. Beyond the income effect: impacts of conditional cash transfer programs on private investments in human capital [Internet]. Washington, DC: World Bank Group; 2014 May p. 111. Report No.: WPS6867. Available: http://documents.worldbank.org/curated/en/2014/05/19520425/beyond-income-effect-impacts-conditional-cash-transfer-programs-private-investments-human-capital

3. Ospina M. The Indirect Effects of Conditional Cash Transfer Programs: An Empirical Analysis of Familias En Accion [Internet]. Dissertation, Georgia State University. 2010. Available: http://scholarworks.gsu.edu/cgi/viewcontent.cgi?article=1059&context=econ_diss
