# Supplementary material for: Comparison of two cash transfer strategies to prevent catastrophic costs for poor tuberculosis-affected households in low- and middle-income countries: An economic modelling study
Source: PLoS Med. 2017 Nov 7;14(11):e1002418. doi: 10.1371/journal.pmed.1002418 (PMC5675360; doi:10.1371/journal.pmed.1002418)
Supplement: S3 Table — The “0.5% of country GDP” column represents the upper limit that governments in low- and middle-income countries spend on a poverty-reduction cash transfer programme [27]. The “poverty-reduction programme” column represents countries’ actual poverty-reduction cash transfer programme budget. The “TB-specific approach” column represents the mean budget that countries would need to prevent their TB-specific target population from incurring catastrophic costs. The “TB-sensitive approach” column represents the mean budget that countries’ would need to prevent their TB-sensitive target population from incurring catastrophic costs. CI, confidence interval; DR, drug-resistant; DS, drug-susceptible; GDP, gross domestic product; TB, tuberculosis. (DOCX) [file pmed.1002418.s005.docx]

|  |  | **Cash transfer budget (2013 PPP$ in millions)** | | | | |
| --- | --- | --- | --- | --- | --- | --- |
| **Country** |  | **0.5% of**  **country GDP** | **Poverty-reduction programme (Range)** | **TB-specific approach**  **(95% CIs) ***† | **TB-sensitive approach**  **(95% CIs) ***† |  |
| **DS TB** |  |  |  |  |  |  |
| Brazil |  | 16,061 | 11,593 (7,835-25,412) | 0.0 (0.0-0.0) | 0.0 (0.0-0.0) |  |
| Ecuador |  | 857 | 485 | 3.8 (3.8-3.8) ‡ | 1,337 (491-2,417) |  |
| Yemen |  | 483 | 1,391 (927-1,546) | 4.4 (4.4-4.5) | 3,424 (2,614-4,228) |  |
| Tanzania |  | 588 | 32 (22-44) | 75 (50-100) | 833 (556-1,106) |  |
| Ghana |  | 518 | 32 (24-46) | 15 (11-19) | 298 (219-378) |  |
| Colombia |  | 3,009 | 2,216 (506-4,705) | 5.9 (5.0-7.3) | 126,352 (93,595-159,037) |  |
| Mexico |  | 10,023 | 6,204 (1,624-13,616) | 50 (41-60) | 165,367 (134,085-196,425) |  |
| **DR TB** |  |  |  |  |  |  |
| Ecuador |  | 857 | 485 | 4.5 (1.6-7.3) | 33,469 (12,072-55,052) |  |

*For interpretability, negative estimates and confidence intervals were reported as 0. †To estimate 95% confidence intervals, all mean TB-related costs were assumed to have a standard deviation with a ratio of 1.1 to their value [1], all mean household incomes were assumed to have a standard deviation with a ratio of 0.8 to their value [2,3], and all mean cash transfers were assumed to have a standard deviation equal to a quarter of maximum minus minimum cash transfers. Probability distributions for all three input parameters were assumed to be normal. This was justified because our analysis was at the national level and we used mean values. ‡Because data were highly skewed we reported median instead of mean.

**References**

1. Tanimura T, Jaramillo E, Weil D, Raviglione M, Lönnroth K. Financial burden for tuberculosis patients in low- and middle-income countries: a systematic review. Eur Respir J. 2014;43: 1763–1775. doi:10.1183/09031936.00193413

2. Cruz M, Ziegelhofer Z. Beyond the income effect: impacts of conditional cash transfer programs on private investments in human capital [Internet]. Washington, DC: World Bank Group; 2014 May p. 111. Report No.: WPS6867. Available: http://documents.worldbank.org/curated/en/2014/05/19520425/beyond-income-effect-impacts-conditional-cash-transfer-programs-private-investments-human-capital

3. Ospina M. The Indirect Effects of Conditional Cash Transfer Programs: An Empirical Analysis of Familias En Accion [Internet]. Dissertation, Georgia State University. 2010. Available: http://scholarworks.gsu.edu/cgi/viewcontent.cgi?article=1059&context=econ_diss
