# Supplementary material for: Comparison of two cash transfer strategies to prevent catastrophic costs for poor tuberculosis-affected households in low- and middle-income countries: An economic modelling study
Source: PLoS Med. 2017 Nov 7;14(11):e1002418. doi: 10.1371/journal.pmed.1002418 (PMC5675360; doi:10.1371/journal.pmed.1002418)
Supplement: S4 Table — The “Before cash transfers” column represents countries’ mean TB-related cost burden without cash transfer data. The “After TB-specific cash transfers” column represents countries’ mean TB-related cost burden after cash transfers have been subtracted from TB-related costs. The “After TB-sensitive cash transfers” column represents countries’ mean TB-related cost burden after cash transfers have been added to countries’ pre-illness household income. CI, confidence interval; DS, drug-susceptible; TB, tuberculosis. (DOCX) [file pmed.1002418.s006.docx]

|  |  | **Cash transfers**  **(% of)** |  | **TB-related cost burden (% of annual household income) *** | | |
| --- | --- | --- | --- | --- | --- | --- |
| **Country** |  | **TB-related costs (95% CIs) §** |  | **Before cash transfers (95% CIs) §** | **After TB-specific cash transfers (95% CIs) §** | **After TB-sensitive cash transfers (95% CIs) §** |
| **DS TB** |  |  |  |  |  |  |
| Brazil† |  | 215 (177-266) |  | 7.3 (5.8-8.7) | 0.0 (0.0-0.0) | 6.3 (5.0-7.6) |
| Tanzania† |  | 28 (23-36) |  | 30 (23-36) | 22 (15-28) | 28 (20-33) |
| Colombia† |  | 119 (102-144) |  | 32 (26-38) | 0.0 (0.0-0.0) | 23 (19-27) |
| Mexico‡ |  | 40 (34-47) |  | 50 (42-58) | 30 (22-38) | 42 (35-49) |

*Household income refers to average household income in the poorest population quintile. †TB-related costs only refer to mean total costs incurred during TB treatment. ‡TB-related costs only refer to mean direct costs. §To estimate 95% confidence intervals, all mean TB-related costs were assumed to have a standard deviation with a ratio of 1.1 to their value [1], all mean household incomes were assumed to have a standard deviation with a ratio of 0.8 to their value [2,3], and all mean cash transfers were assumed to have a standard deviation equal to a quarter of maximum minus minimum cash transfers.

**References**

1. Tanimura T, Jaramillo E, Weil D, Raviglione M, Lönnroth K. Financial burden for tuberculosis patients in low- and middle-income countries: a systematic review. Eur Respir J. 2014;43: 1763–1775. doi:10.1183/09031936.00193413

2. Cruz M, Ziegelhofer Z. Beyond the income effect: impacts of conditional cash transfer programs on private investments in human capital [Internet]. Washington, DC: World Bank Group; 2014 May p. 111. Report No.: WPS6867. Available: http://documents.worldbank.org/curated/en/2014/05/19520425/beyond-income-effect-impacts-conditional-cash-transfer-programs-private-investments-human-capital

3. Ospina M. The Indirect Effects of Conditional Cash Transfer Programs: An Empirical Analysis of Familias En Accion [Internet]. Dissertation, Georgia State University. 2010. Available: http://scholarworks.gsu.edu/cgi/viewcontent.cgi?article=1059&context=econ_diss
