# Supplementary material for: Comparison of two cash transfer strategies to prevent catastrophic costs for poor tuberculosis-affected households in low- and middle-income countries: An economic modelling study
Source: PLoS Med. 2017 Nov 7;14(11):e1002418. doi: 10.1371/journal.pmed.1002418 (PMC5675360; doi:10.1371/journal.pmed.1002418)
Supplement: S5 Table — The “additional cash transfer” column represents the additional value of cash transfer that countries’ average TB-affected household would need to prevent catastrophic costs using a TB-specific versus a TB-sensitive approach. The “total cash transfer” column represents the total value that countries’ average TB-affected household would need to prevent catastrophic costs using a TB-specific versus a TB-sensitive approach. The “cash transfer budget, in millions” column represents the mean budget that countries would need to prevent catastrophic costs for its TB-specific versus TB-sensitive target populations. CI, confidence interval; DS, drug-susceptible; PPP, purchasing power parity; TB, tuberculosis. (DOCX) [file pmed.1002418.s007.docx]

|  |  | **Additional cash transfer**  **2013 PPP$ (95% CIs) ‡** | |  | **Total cash transfer,**  **2013 PPP$ (95% CIs) ‡** | |  | **Cash transfer budget,**  **2013 PPP$ in millions (95% CIs) ‡** | | |
| --- | --- | --- | --- | --- | --- | --- | --- | --- | --- | --- |
| **Country** |  | **TB-specific approach** | **TB-sensitive approach** |  | **TB-specific approach** | **TB-sensitive approach** |  | **TB-specific**  **approach** | **TB-sensitive approach** |  |
| **DS TB** |  |  |  |  |  |  |  |  |  |  |
| Brazil* |  | 0.0  (0.0-0.0) | 0.0  (0.0-0.0) |  | 0.0  (0.0-0.0) | 0.0  (0.0-0.0) |  | 0.0  (0.0-0.0) | 0.0  (0.0-0.0) |  |
| Tanzania* |  | 63  (0.0-225) | 1,136  (209-2,063) |  | 294  (229-459) | 1,367  (441-2,294) |  | 20  (16-31) | 205  (66-344) |  |
| Colombia* |  | 0.0  (0.0-0.0) | 495  (0.0-1,097) |  | 837  (823-851) | 1,332  (835-1,934) |  | 5.0  (4.9-5.1) | 34,637  (21,713-50,288) |  |
| Mexico† |  | 490  (109-870) | 6,208  (4,314-8,110) |  | 1,430  (1,050-1,810) | 7,148  (5,252-9,048) |  | 14  (11-18) | 47,176  (34,660-59,717) |  |

*TB-related costs only refer to mean total costs incurred during TB treatment. †TB-related costs only refer to mean direct costs. ‡To estimate 95% confidence intervals, all mean TB-related costs were assumed to have a standard deviation with a ratio of 1.1 to their value [1], all mean household incomes were assumed to have a standard deviation with a ratio of 0.8 to their value [2,3], and all mean cash transfers were assumed to have a standard deviation equal to a quarter of maximum minus minimum cash transfers.

**References**

1. Tanimura T, Jaramillo E, Weil D, Raviglione M, Lönnroth K. Financial burden for tuberculosis patients in low- and middle-income countries: a systematic review. Eur Respir J. 2014;43: 1763–1775. doi:10.1183/09031936.00193413

2. Cruz M, Ziegelhofer Z. Beyond the income effect: impacts of conditional cash transfer programs on private investments in human capital [Internet]. Washington, DC: World Bank Group; 2014 May p. 111. Report No.: WPS6867. Available: http://documents.worldbank.org/curated/en/2014/05/19520425/beyond-income-effect-impacts-conditional-cash-transfer-programs-private-investments-human-capital

3. Ospina M. The Indirect Effects of Conditional Cash Transfer Programs: An Empirical Analysis of Familias En Accion [Internet]. Dissertation, Georgia State University. 2010. Available: http://scholarworks.gsu.edu/cgi/viewcontent.cgi?article=1059&context=econ_diss
