# Supplementary material for: Comparison of two cash transfer strategies to prevent catastrophic costs for poor tuberculosis-affected households in low- and middle-income countries: An economic modelling study
Source: PLoS Med. 2017 Nov 7;14(11):e1002418. doi: 10.1371/journal.pmed.1002418 (PMC5675360; doi:10.1371/journal.pmed.1002418)
Supplement: S6 Table — The “additional cash transfer” column represents the additional value of cash transfer that countries’ average TB-affected household would need to prevent catastrophic costs using a TB-specific versus a TB-sensitive approach. The “total cash transfer” column represents the total value that countries’ average TB-affected household would need to prevent catastrophic costs using a TB-specific versus a TB-sensitive approach. The “cash transfer budget, in millions” column represents the mean budget that countries would need to prevent catastrophic costs for their TB-specific versus TB-sensitive target populations. CI, confidence interval; DR, drug-resistant; DS, drug-susceptible; PPP, purchasing power parity; TB, tuberculosis. (DOCX) [file pmed.1002418.s008.docx]

|  |  | **Additional cash transfer,**  **2013 PPP$ (95% CIs) *** | |  | **Total cash transfer,**  **2013 PPP$ (95% CIs) *** | |  | | **Cash transfer budget,**  **2013 PPP$ in millions (95% CIs) *** | |
| --- | --- | --- | --- | --- | --- | --- | --- | --- | --- | --- |
| **Country** |  | **TB-specific approach** | **TB-sensitive approach** |  | **TB-specific approach** | **TB-sensitive approach** |  | **TB-specific approach** | | **TB-sensitive approach** |
| **DS TB** |  |  |  |  |  |  |  |  | |  |
| Brazil |  | 0.0  (0.0-0.0) | 1,595  (32-3,156) |  | 823  (815-831) | 2,424  (855-3,976) |  | 29  (28-29) | | 33,939  (11,976-55,659) |
| Ecuador |  | 365  (0.0-854) | 13,467  (8,593-18,360) |  | 1,464  (1,091-1,945) | 14,558  (9,684-19,451) |  | 5.2  (3.8-6.8) | | 6,551 (4,358-8,753) |
| Yemen |  | 0.0  (0.0-0.0) | 5,786 (4,711-6,856) |  | 923  (920-926) | 6,709  (5,632-7,779) |  | 4.4  (4.4-4.5) | | 10,064  (8,448-11,669) |
| Tanzania |  | 1,175  (805-1,540) | 13,701  (10,006-17,346) |  | 1,392  (1,022-1,756) | 13,918  (10,223-17,562) |  | 94 (69-119) | | 2,088  (1,533-2,634) |
| Ghana |  | 578  (355-806) | 9,835  (7,609-12,118) |  | 1,029  (806-1,257) | 10,286  (8,060-12,569) |  | 18 (14-22) | | 720  (564-880) |
| Colombia |  | 356  (106-608) | 11,096 (8,579-13,618) |  | 1,193  (942-1,445) | 11,933  (9,417-14,448) |  | 7.2 (5.7-8.7) | | 310,268  (244,845-375,659) |
| Mexico |  | 4,547  (3,597-5,490) | 53,926 (44,445-63,345) |  | 5,487  (4,539-6,428) | 54,866  (45,387-64,281) |  | 55 (45-64) | | 362,118  (299,555-424,258) |
| **DR TB** |  |  |  |  |  |  |  |  | |  |
| Ecuador |  | 14,652  (5,142-24,241) | 156,334  (61,239-252,232) |  | 15,744  (6,233-25,332) | 157,435 (62,330-253,323) |  | 4.7 (1.9-7.6) | | 70,846 (28,049-113,995) |

*To estimate 95% confidence intervals, all mean TB-related costs were assumed to have a standard deviation with a ratio of 1.1 to their value [1], all mean household incomes were assumed to have a standard deviation with a ratio of 0.8 to their value [2,3], and all mean cash transfers were assumed to have a standard deviation equal to a quarter of maximum minus minimum cash transfers.

**References**

1. Tanimura T, Jaramillo E, Weil D, Raviglione M, Lönnroth K. Financial burden for tuberculosis patients in low- and middle-income countries: a systematic review. Eur Respir J. 2014;43: 1763–1775. doi:10.1183/09031936.00193413

2. Cruz M, Ziegelhofer Z. Beyond the income effect: impacts of conditional cash transfer programs on private investments in human capital [Internet]. Washington, DC: World Bank Group; 2014 May p. 111. Report No.: WPS6867. Available: http://documents.worldbank.org/curated/en/2014/05/19520425/beyond-income-effect-impacts-conditional-cash-transfer-programs-private-investments-human-capital

3. Ospina M. The Indirect Effects of Conditional Cash Transfer Programs: An Empirical Analysis of Familias En Accion [Internet]. Dissertation, Georgia State University. 2010. Available: http://scholarworks.gsu.edu/cgi/viewcontent.cgi?article=1059&context=econ_diss
