# Supplementary material for: Comparison of two cash transfer strategies to prevent catastrophic costs for poor tuberculosis-affected households in low- and middle-income countries: An economic modelling study
Source: PLoS Med. 2017 Nov 7;14(11):e1002418. doi: 10.1371/journal.pmed.1002418 (PMC5675360; doi:10.1371/journal.pmed.1002418)
Supplement: S7 Table — The “additional cash transfer” column represents the additional value of cash transfer that countries’ average TB-affected household would need to prevent catastrophic costs using a TB-specific versus a TB-sensitive approach. The “total cash transfer” column represents the total value that countries’ average TB-affected household would need to prevent catastrophic costs using a TB-specific versus a TB-sensitive approach. The “cash transfer budget, in millions” column represents the mean budget that countries’ would need to prevent catastrophic costs for their TB-specific versus TB-sensitive target populations. CI, confidence interval; DR, drug-resistant; DS, drug-susceptible; PPP, purchasing power parity; TB, tuberculosis. (DOCX) [file pmed.1002418.s009.docx]

|  |  | **Additional cash transfer,**  **2013 PPP$ (95% CIs) *** | |  | **Total cash transfer,**  **2013 PPP$ (95% CIs) *** | |  | **Cash transfer budget,**  **2013 PPP$ in millions (95% CIs) *** | | |
| --- | --- | --- | --- | --- | --- | --- | --- | --- | --- | --- |
| **Country** |  | **TB-specific approach** | **TB-sensitive approach** |  | **TB-specific approach** | **TB-sensitive approach** |  | **TB-specific approach** | **TB-sensitive approach** |  |
| **DS TB** |  |  |  |  |  |  |  |  |  |  |
| Brazil |  | 0.0  (0.0-0.0) | 0.0  (0.0-0.0) |  | 0.0  (0.0-0.0) | 0.0  (0.0-0.0) |  | 0.0  (0.0-0.0) | 0.0  (0.0-0.0) |  |
| Ecuador |  | 0.0  (0.0-0.0) | 0.0  (0.0-0.0) |  | 0.0  (0.0-0.0) | 0.0  (0.0-0.0) |  | 0.0  (0.0-0.0) | 0.0  (0.0-0.0) |  |
| Yemen |  | 0.0  (0.0-0.0) | 0.0 (0.0-244) |  | 923  (920-926) | 952  (920-1,167) |  | 4.4  (4.4-4.5) | 1,428  (1,380-1,751) |  |
| Tanzania |  | 612  (242-977) | 2,548  (1,314-3,760) |  | 829  (460-1,193) | 2,765  (1,533-3,978) |  | 56 (31-81) | 415  (230-597) |  |
| Ghana |  | 221  (0-449) | 1,788  (1,045-2,551) |  | 673  (451-900) | 2,239  (1,496-3,002) |  | 12 (7.9-16) | 157 (105-210) |  |
| Colombia |  | 0.0 (0.0-167) | 1,665  (826-2,504) |  | 856  (823-1,002) | 2,502  (1,661-3,339) |  | 5.1  (4.9-6.0) | 65,046  (43,188-86,807) |  |
| Mexico |  | 3,596 (2,646-4,538) | 14,179  (11,015-17,317) |  | 4,536  (3,587-5,477) | 15,119  (11,958-18,258) |  | 45 (36-55) | 99,784  (78,920-120,501) |  |
| **DR TB** |  |  |  |  |  |  |  |  |  |  |
| Ecuador |  | 12,913  (3,410-22,512) | 45,589  (13,911-77,584) |  | 14,010  (4,501-23,603) | 46,692  (15,002-78,675) |  | 4.2 (1.4-7.1) | 21,011  (6,751-35,404) |  |

*To estimate 95% confidence intervals, all mean TB-related costs were assumed to have a standard deviation with a ratio of 1.1 to their value [1], all mean household incomes were assumed to have a standard deviation with a ratio of 0.8 to their value [2,3], and all mean cash transfers were assumed to have a standard deviation equal to a quarter of maximum minus minimum cash transfers.

**References**

1. Tanimura T, Jaramillo E, Weil D, Raviglione M, Lönnroth K. Financial burden for tuberculosis patients in low- and middle-income countries: a systematic review. Eur Respir J. 2014;43: 1763–1775. doi:10.1183/09031936.00193413

2. Cruz M, Ziegelhofer Z. Beyond the income effect: impacts of conditional cash transfer programs on private investments in human capital [Internet]. Washington, DC: World Bank Group; 2014 May p. 111. Report No.: WPS6867. Available: http://documents.worldbank.org/curated/en/2014/05/19520425/beyond-income-effect-impacts-conditional-cash-transfer-programs-private-investments-human-capital

3. Ospina M. The Indirect Effects of Conditional Cash Transfer Programs: An Empirical Analysis of Familias En Accion [Internet]. Dissertation, Georgia State University. 2010. Available: http://scholarworks.gsu.edu/cgi/viewcontent.cgi?article=1059&context=econ_diss
